# Origin and Distribution of the Brachial Plexus in Two Procyonids (*Procyon cancrivorus* and *Nasua nasua*, Carnivora)

**DOI:** 10.3390/ani13020210

**Published:** 2023-01-06

**Authors:** Juan Fernando Vélez García, Roseãmely Angélica de Carvalho Barros, Maria Angélica Miglino

**Affiliations:** 1 Department of Animal Health, Faculty of Veterinary Medicine and Zootechnics, Universidad del Tolima, Ibague 730006299, Colombia; 2 Department of Surgery, Faculty of Veterinary Medicine and Animal Science, Universidade de São Paulo, Butantã CEP 05508-270, Brazil; 3 Comparative Animal Laboratory, Biological Sciences Department, Biotechnology Institute, Universidade Federal de Catalão, Catalão 75704-020, Brazil

**Keywords:** anatomy, ansa axillaris, Caniformia, communicating branches, nerve, neurology

## Abstract

**Simple Summary:**

The brachial plexus is a nervous network from which nerves originate to innervate the thoracic limb and its adjacent parts. It has been found with different origins and distributions in the thoracic limb between carnivoran species (interspecific) and even in the same species (intraspecific). Anatomical knowledge of the brachial plexus allows us to understand the differences and similarities between species to be applied in evolutionary biology. *Procyon cancrivorus* and *Nasua nasua* are two neotropical carnivoran species that have different evolutionary adaptations in their thoracic limbs. The aim of this study was to describe the anatomical arrangement of the brachial plexus of these species to review intra- and interspecific anatomical variations. The brachial plexus may originate in both species from the last four cervical spinal nerves (C5, C6, C7 and C8) and the first two thoracic spinal nerves (T1 and T2). The contribution from C5 was higher in *Nasua nasua*, while the contribution from T2 was higher in *Procyon cancrivorus*. Interestingly, both species develop ansa axillaris and ansa pectoralis, which is a primitive anatomical arrangement that is shared with other members of arctoid carnivorans. Therefore, this study confirmed that both structures can be present in some wild carnivorans.

**Abstract:**

*Procyon cancrivorus* and *Nasua nasua* are two procyonids with different evolutionary adaptations to use their thoracic limbs. Therefore, this study aimed to characterize the differences in the brachial plexus between both species. Five *P. cancrivorus* and five *N. nasua* cadavers were used to perform this investigation with the permission of the bioethics committee and environmental license. Gross dissections were performed on the cervical, pectoral, and thoracic limb regions to find the origin and distribution of the brachial plexus. The brachial plexus of both species originated in a variant manner from C5-T1, C5-T2, C6-T1, or C6-T2. All brachial plexus nerves were observed and, interestingly, the musculocutaneous sent a communicating branch to the median nerve medially to the axillary artery, forming an ansa axillaris in both species. An ansa pectoralis was also observed medially to the axillary artery. Additionally, in *P. cancrivorus*, the musculocutaneous nerve innervates the pronator teres and flexor carpi radialis muscles and communicates with the median nerve at the elbow level to continue as a common trunk at the antebrachium. The brachial plexus has differences between both procyonids, although in both species, it could conserve a primitive arrangement present within the infraorder Arctoidea.

## 1. Introduction

The crab-eating raccoon (*Procyon cancrivorus*) and the coati (*Nasua nasua*) are two mammal species that belong to the order Carnivora, suborder Caniformia, and family Procyonidae [1]. *P. cancrivorus* is geographically distributed in Central and South America [2], while *N. nasua* is only distributed in South America [3]. The genera *Procyon* and *Nasua* had a common ancestor within the family Procyonidae [1,4], which diverged approximately 17 million years ago [1]. *P. cancrivorus* has crepuscular, nocturnal, and solitary habits [5,6], while *N. nasua* is gregarious with diurnal habits, although adult males have mainly solitary habits [7].

The *P. cancrivorus* diet is based on fruits, crabs, mollusks, arthropods, and small vertebrates [8,9]. The diet of *N. nasua* is mainly composed of fruits and ground invertebrates [7,10,11], although it is also an opportunistic hunter of small vertebrates and sometimes eats eggs [7,10,12,13]. Both species are mainly terrestrial species [7]; however, *N. nasua* sometimes has arboreal preferences [11] and uses trees to rest, escape predators, or nest [11,14,15]. Both species are good climbers [16], although *N. nasua* uses its hands mainly to excavate (fossorial abilities) and shred dead logs [5,11,17]. *Procyon,* due to the well-developed sense of touch in its hands, uses them to locate and handle animal prey [5,17], manipulating the food to wash it and put it in the mouth [16,18]. In addition, *Procyon* is also a good swimmer and fisher [16].

*Procyon* has long thoracic limbs adapted to semidigitigrade and cursorial locomotion, while *Nasua* has short thoracic limbs adapted to palmigrade locomotion with long claws [7,17]. *Nasua* has an enlarged sensory cortex region for receiving the afferent projections from the vibrissae of the nose and tactile carpal hairs, and *Procyon* has a well-developed cerebral sensorimotor cortex, which is focused in its hands due to the high density of cutaneous nerves [5]. Therefore, the brachial plexus and its nerves could have evolved in different forms in both procyonid species, since they are responsible for the sensitivity and movements of the thoracic limb. In addition, specific knowledge about the brachial plexus could be used for medical and surgical procedures and locoregional anesthesia in wild carnivorans [19].

Some brachial plexus studies in wild carnivorans only reported the origin of the nerves [20,21,22], while others reported the origin and distribution of the nerves up to the antebrachium [23,24,25,26,27] and others up to the manus [24,28,29,30,31]. However, among these latter studies, only two report innervation of the intrinsic muscles of the manus [24,30]. In most carnivoran species, the brachial plexus originates from the ventral branches of the last three cervical spinal nerves and the first thoracic spinal nerve (C6-T1). The contributions of the fifth cervical spinal nerve (C5) and the second thoracic spinal nerve (T2) have been found in few carnivorans. However, C5 has been found in a high proportion in some felids [23,32], and T2 in some musteloids [30,31]. Therefore, the aim of this study was to describe the origin and distribution of the brachial plexus along the thoracic limb in *P. cancrivorus* and *N. nasua*.

## 2. Materials and Methods

### 2.1. Specimens

Five cadavers of *P. cancrivorus* (three adult males and two juvenile females) (Table A1) and five of *N. nasua* (two adult males, one juvenile male, one infant male, and one adult female) were used to perform this investigation (Table A2). Two *P. cancrivorus* (one male and one female) and one *N. nasua* were donated by CORPOCALDAS (Environmental authority of Caldas, Colombia) to the Universidad del Tolima. One *P. cancrivorus* was donated by CORTOLIMA (Environmental authority of Tolima, Colombia). Four *N. nasua* and one *P. cancrivorus* were collected from roads to the Universidade Federal de Catalão with a license from the Ministry of Environment of Brazil (SISBIO Number 37072-2). Three *P. cancrivorus* and four *N. nasua* had died from being run over on roads, and the other specimens died of natural causes. The bioethics committees of the Universidad del Tolima (agreement number 2.3-059), Universidade de São Paulo (CEUAx agreement number 3928240820), and Universidade de Catalão (CEUA-UFCAT agreement number 01/22) approved the use of these specimens in this investigation.

### 2.2. Fixation and Vascular Repletion of Specimens

The specimens were fixed with a solution of 10% formaldehyde via the femoral artery and were posteriorly immersed and conserved in closed containers with 5% formaldehyde. One *P. cancrivorus* and all *N. nasua* specimens were repleted with natural latex tinctured with red vinyl via the femoral artery. One *P. cancrivorus* was repleted via the axillary artery due to a previous necropsy. The other specimens were not replete.

### 2.3. Dissection and Documentation

Gross dissections were performed at the Amphitheater of Veterinary Anatomy of the Universidad del Tolima (Tolima, Colombia) and Comparative Anatomy of Wild Animals Laboratory of the Universidade Federal de Catalão (Goias, Brazil). First, the skin was removed from the dorsal regions of the neck and trunk to the distal extreme of the thoracic limbs. The fascia around the muscles, nerves, and vessels was removed to review the distribution of the nerves. The pectoral muscles were disinserted from the sternum and the ventral part of the thoracic wall was removed to review the vascular relationships of the brachial plexus. Finally, the vessels, viscera, and ventral muscles of the neck and thorax were removed until the emergence of the ventral branches of the cervical and thoracic spinal nerves was found. The anatomical descriptions were performed according to the terminology of the *Nomina Anatomica Veterinaria* -*NAV*- [33]. However, other terms that do not appear in the *NAV* were used due to the presence of other nerves and muscles in carnivorans, such as n. brachiocephalicus [34], n. teres major [30,35], m. pectoralis abdominalis [36,37], m. anconeus medialis [38], and m. palmaris longus [39]. Photographs of the dissections were taken with a Canon EOS 6D 20.2 MP fitted with a 100 mm Canon macro lens and a Canon T5i 18 MP camera fitted with a 50 mm Canon macro lens. The origin of each brachial plexus nerve was expressed in percentage values.

## 3. Results

### 3.1. Origin of the Brachial Plexus in Procyon cancrivorus and Nasua nasua

The brachial plexus in four specimens of *P. cancrivorus* originates bilaterally from the ventral branches of the cervical spinal nerves C6, C7, and C8, and the thoracic spinal nerves T1 and T2 (C6-T2). In one specimen, it originates from C5-T1 bilaterally (Pc5). In *N. nasua*, the brachial plexus originates from C5-T2 in six limbs, C5-T1 in two limbs, and C6-T1 in two limbs.

**Figure 1:**
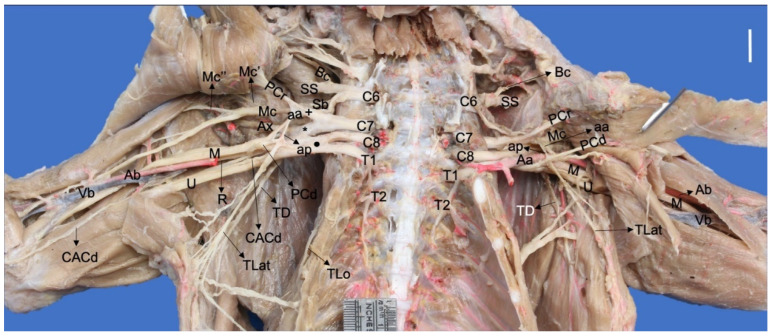
Ventral photographic view of the brachial plexus in a *Procyon cancrivorus*. (Aa) axillary artery, (aa) ansa axillaris (proximal communicating branch to the median and caudal pectoral nerves), (Ab) brachial artery, (ap) ansa pectoralis (communicating branch to the nn. Pectorales caudales), (Ax) n. axillaris, (Bc) n. brachiocephalicus, (C6–8) cervical spinal nerves, (CACd) n. cutaneus antebrachii caudalis, (M) n. medianus, (Mc) n. musculocutaneus, (Mc′) branch to the m. coracobrachialis, (Mc″) ramus muscularis proximalis, (PCr) nn. Pectorales craniales, (PCd) nn. Pectorales caudales, (R) n. radialis, (Sb) nn. subscapulares, (SS) n. suprascapularis, (T1–T2) thoracic spinal nerves, (TD) n. thoracodorsalis, (TLat) n. thoracicus lateralis, (TLo) n. thoracicus longus, (U) n. ulnaris, (Vb) brachial vein, (+) cranial ventral trunk, (*) cranial dorsal trunk, (black circle) caudal ventral trunk. White bar: 10 mm.

The branch of C5 to the brachial plexus is a small branch that extends parallel to the longus capitis muscle until reaching the ventral branch of C6. The ventral branch of C6 emerges between the longus capitis and the scalenus muscles, while the ventral branches of C7 to T1 emerge between the longus colli and the scalenus muscles. T2 joins with T1 in the medial surface of the first rib before it emerges toward the axillary space, which is a small branch. In one *P. cancrivorus* (Pc2), T2 is a large branch bilaterally. The ventral branches of C5, C6, C7, and C8 have a ventral relationship with the external jugular vein and the superficial cervical artery, while the common trunk of T1 and T2 has a ventral relationship with the axillary artery. C6 crosses ventrally to the scalenus muscle and sends two large branches (cranial and caudal), with the cranial directed laterally to form the suprascapular and brachiocephalic nerves. The caudal branch extends parallel to the scalenus muscle, forms the cranial subscapular nerve, and joins the ventral branch of C7. When C5 contributes to the brachial plexus, it joins C6 and forms the brachiocephalicus, suprascapular, and cranial subscapular nerves in *N. nasua,* and only the two former nerves in *P. cancrivorus* (Figure 1).

**Figure 2:**
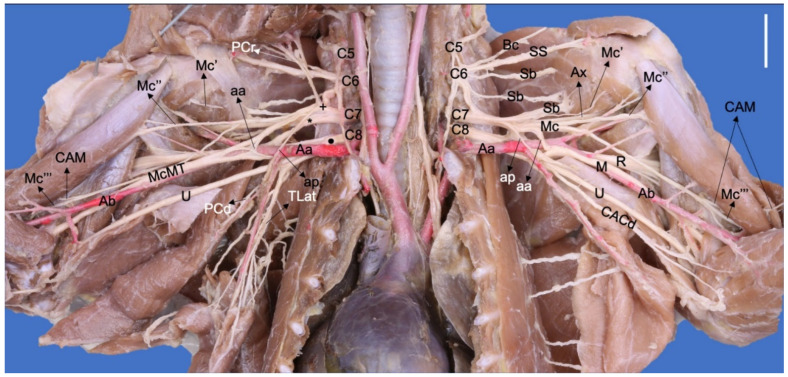
Ventral photographic view of the brachial plexus with arterial relationships in a *Nasua nasua*. (Aa) axillary artery, (aa) ansa axillaris (proximal communicating branch to the n. medianus), (Ab) brachial artery, (ap) ansa pectoralis (communicating branch to the nn. pectorales caudales), (Ax) n. axillaris, (Bc) n. brachiocephalicus, (Bv) brachial vein, (C5–8) cervical spinal nerves, (CACd) n. cutaneus antebrachii caudalis, (CAM) n. cutaneus antebrachii medialis, (M) n. medianus, (Mc) n. musculocutaneus, (Mc′) branch to coracobrachialis, (Mc″) ramus muscularis proximalis, (Mc‴) ramus muscularis distalis, (PCr) nn. pectorales craniales, (PCd) nn. pectorales caudales, (R) n. radialis, (Sb) nn. subscapulares, (SS) n. suprascapularis, (TD) n. thoracodorsalis, (TLat) n. thoracicus lateralis, (TLo) n. thoracicus longus, (McMT) Mc and M common trunk, (U) n. ulnaris, (Vb) brachial vein, (+) cranial ventral trunk, (*) cranial dorsal trunk, (black circle) caudal ventral trunk. White bar: 10 mm.

The ventral branch of C7 forms two large branches, one dorsal and one ventral, where both receive branches from the caudal branch of C6 and form two trunks, dorsal and ventral, respectively. The ventral cranial trunk forms the musculocutaneous and cranial pectoral nerves. In two *N. nasua* (Nn1 and Nn5), it also forms an ansa pectoralis, which is a communicating branch with the caudal pectoral nerves (Figure 2). In the other three *N. nasua*, C7 directly forms the ansa pectoralis, and in one case, it also communicates with the lateral thoracic nerve (Nn5). In the case of *P. cancrivorus*, the ansa axillaris forms the ansa pectoralis (Figure 1).

**Figure 3:**
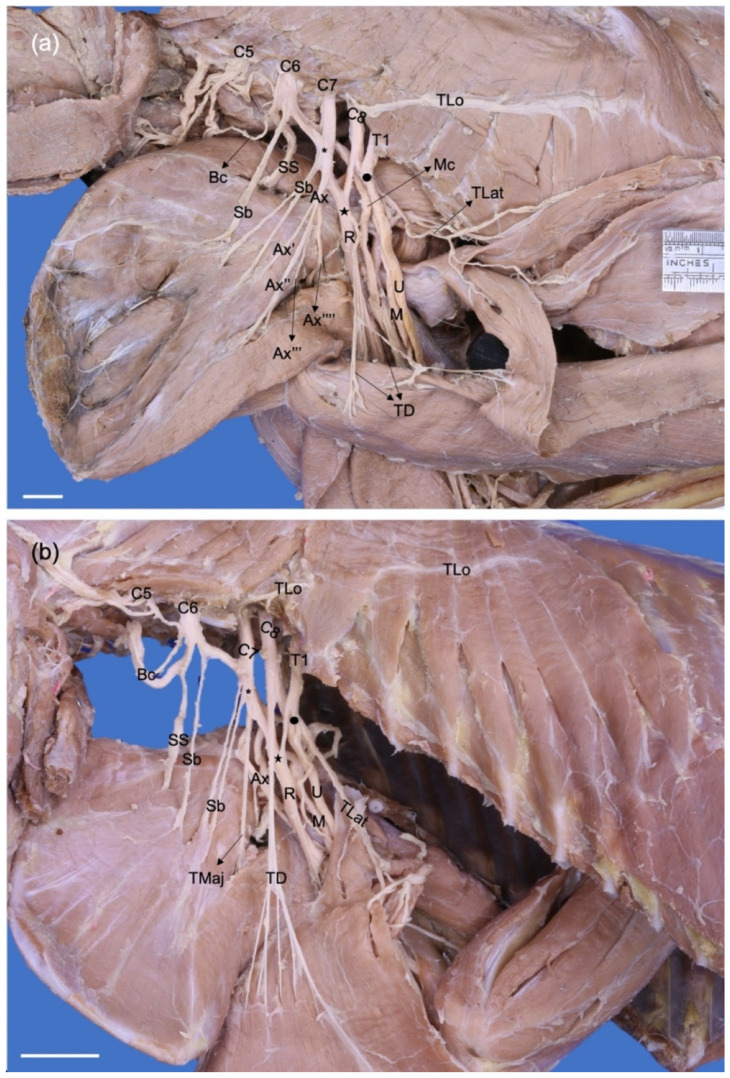
Dorsolateral photographic views of left brachial plexuses of a (**a**) *Procyon cancrivorus* and (**b**) *Nasua nasua*. (Ax) n. axillaris, (Ax′) branch to the m. subscapularis, (Ax″) branch to the teres major and subscapularis muscles, (Ax‴) branch to the m. teres minor, (Ax⁗) branch to the deltoideus muscles and n. cutaneus brachii cranialis, (Bc) n. brachiocephalicus, (C6–8) cervical spinal nerves, (M) n. medianus, (Mc) n. musculocutaneus, (R) n. radialis, (Sb) nn. subscapulares, (SS) n. suprascapularis, (T1) first thoracic spinal nerve, (TD) n. thoracodorsalis, (TLat) n. thoracicus lateralis, (TLo) n. thoracicus longus, (TMaj) n. teres major, (U) n. ulnaris, (*) cranial dorsal trunk, (black star) caudal dorsal trunk, (black circle) caudal ventral trunk. White bars: 10 mm.

The dorsal cranial trunk forms the caudal subscapular and axillary nerves. It also forms an independent nerve directed to the m. teres major (*n. teres major*) and the m. subscapularis in all cases of *N. nasua* and one case bilaterally in *P. cancrivorus* (Nn5). The ventral branches of C8, T1, and T2 form two common trunks just lateral to the m. scalenus, the ventral caudal and dorsal caudal trunks. The former forms the common trunk of the median and ulnar nerves and a common trunk to the lateral thoracic and caudal pectoral nerves (Figure 1). The dorsal caudal trunk joins with a branch from the dorsal cranial trunk and forms the radial and thoracodorsal nerves (Figure 3).

### 3.2. Origin and Distribution of the Brachial Plexus Nerves in Procyon cancrivorus and Nasua nasua

The origin and distribution of each nerve are summarized in Table 1.

**Figure 4:**
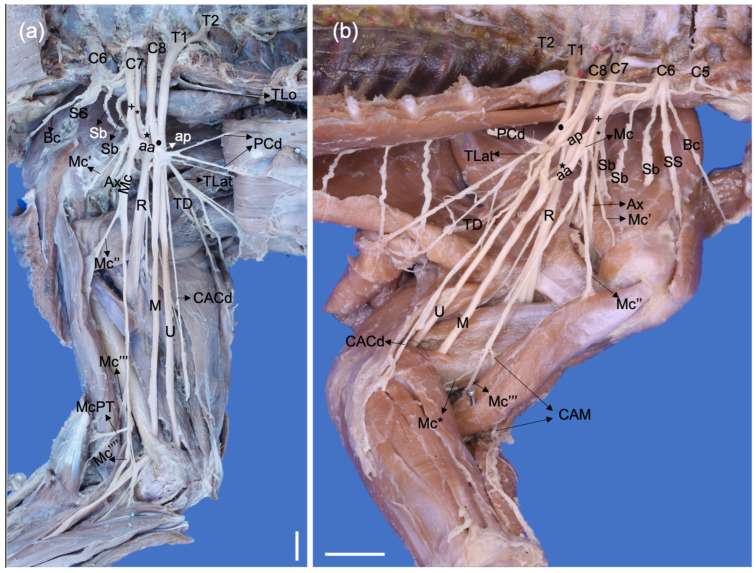
Medial photographic views of a right brachial plexus of a *Procyon cancrivorus* (**a**) and a left brachial plexus of a *N. nasua* (**b**). (aa) ansa axillaris (proximal communicating branch of the Mc to M), (ap) ansa pectoralis, (Ax) n. axillaris, (Bc) n. brachiocephalicus, (C6–8) cervical spinal nerves, (CACd) n. cutaneus antebrachii caudalis, (CAM) n. cutaneus antebrachii medialis, (M) n. medianus, (Mc) n. musculocutaneus, (Mc′) branch to coracobrachialis, (Mc″) ramus muscularis proximalis, (Mc‴) ramus muscularis distalis, (Mc⁗) distal communicating branch with the median nerve, (Mc*) branch to the joint capsule of the elbow, (McPT) branch to the m. pronator teres, (PCr) nn. pectorales craniales, (PCd) nn. pectorales caudales, (R) n. radialis, (Sb) nn. subscapulares, (SS) n. suprascapularis, (T1-2) thoracic spinal nerves, (TD) n. thoracodorsalis, (TLat) n. thoracicus lateralis, (TLo) n. thoracicus longus, (U) n. ulnaris, (+) cranial ventral trunk, (*) cranial dorsal trunk, (black star) caudal dorsal trunk, (black circle) caudal ventral trunk. White bars: 10 mm.

The brachiocephalic (*N. brachiocephalicus*) and suprascapular nerves (*N. suprascapularis*) are formed from a single trunk in all *P. cancrivorus* and one limb of *N. nasua* (Nn1), while both originate independently in most limbs of *N. nasua* (Figure 1, Figure 2 and Figure 3). The brachiocephalic nerve extends laterally toward the space between the omotransversarius and cleidocephalicus muscles, passing superficially to the m. cleidobrachialis. In two *P. cancrivorus* (Pc3 and Pc4), the nerve receives communication from the ventral branch of C5 bilaterally just before passing through the space toward the skin. It innervates the cranial regions of the shoulder and the proximal half of the brachium and does not send a muscle branch to the m. cleidobrachialis. In one *P. cancrivorus*, it also communicates bilaterally with the cranial lateral brachial cutaneous nerve (Pc4). In one limb of *P. cancrivorus* (Pc5), the n. brachiocephalicus perforates the m. supraspinatus, innervates it, and continues laterally along its normal course. The suprascapular nerve passes between the subscapularis and supraspinatus muscles, cranially to the scapular incisure and laterally to the scapular neck until the m. infraspinatus. Two subscapular nerves (*Nn. subscapulares*) are formed in *P. cancrivorus*, and three are formed in *N. nasua.* However, three nerves are formed in one limb of *P. cancrivorus* (Pc4) and two nerves bilaterally in *N. nasua* (Nn4) (Figure 4).

The axillary nerve (*N. axillaris*) first sends one branch to the most caudal belly of the m. subscapularis and, in most cases, to the m. teres major in *P. cancrivorus*. In all *N. nasua* and one *P. cancrivorus* (Pc5), the nerve to the teres major muscle (*N. teres major*) originates directly from the dorsal cranial trunk and sends a small branch to the m. subscapularis (Figure 3). The axillary nerve passes between the subscapular and teres major muscles to continue laterally deeply to the m. deltoideus, sending branches to the m. teres minor, and acromial and scapular parts of the m. deltoideus and m. cleidobrachialis. Deep in the m. deltoideus, it forms the cranial lateral brachial cutaneous nerve (*N. cutaneus brachii lateralis cranialis*), which extends between the m. brachialis and acromial part of the m. deltoideus to innervate the distal half of the brachium and elbow. It communicates with the medial branch of the superficial branch of the radial nerve in *N. nasua* and directly with the superficial branch of the radial nerve in *P. cancrivorus*. However, in one limb of *N. nasua* (Nn1), it also communicates with the lateral branch of the superficial radial nerve.

The radial nerve (N. *radialis*) extends laterally to the axillary and brachial arteries. Medial to the common tendon of the latissimus dorsi and teres major muscles, it forms branches to the m. tensor fasciae antebrachii, capita longum, mediale, and accessorium of the m. triceps brachii. The nerve passes laterally between the capita accessorium and mediale of the m. triceps brachii at the proximal third of the brachium (Figure 4). It continues laterally at the brachium between the m. brachialis and the caput laterale of m. triceps brachii, sending a branch to this caput and another branch to the m. anconeus. At this level, the nerve divides into deep and superficial branches, where the former innervates the craniolateral antebrachial muscles, and the latter innervates the craniolateral surface of the antebrachium and dorsum of the manus. The deep branch (*Ramus profundus*) first innervates the brachioradialis and extensor carpi radialis muscles and passes deeply to the m. supinator to innervate the other muscles. It also innervates the m. brachialis unilaterally in two cases in *P. cancrivorus* (Pc2 and Pc5).

**Figure 5:**
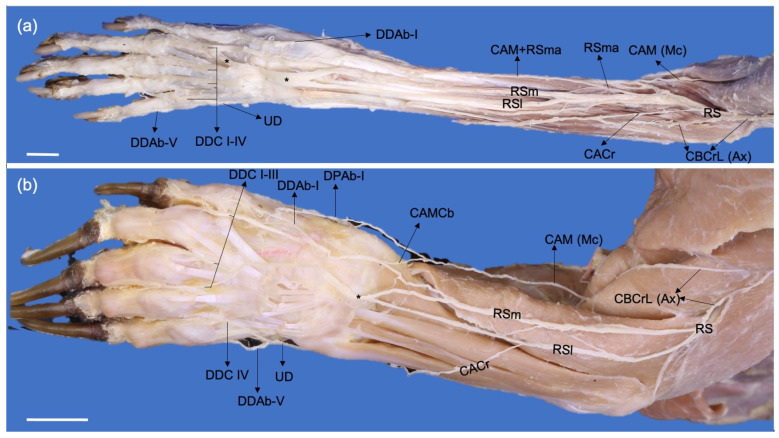
Cranial photographic views of the left antebrachia of a *P. cancrivorus* (**a**) and a *N. nasua* (**b**). (Ax) n. axillaris, (CACr) n. cutaneus antebrachii cranialis, (CAM) n. cutaneus antebrachii medialis, (CAMCb) communicating branch from CAM to RSm, (CBCrL) n. cutaneus brachii cranialis lateralis, (DDAb-I) n. digitalis dorsalis I abaxialis, (DDAb-V) n. digitalis dorsalis V abaxialis, (DDC I-IV) nn. digitales dorsales communes I-IV, (DPAb-I) n. digitalis palmaris I abaxialis, (Mc) n. musculocutaneus, (RS) superficial branch of the n. radialis, (RSl) lateral branch, (RSm) medial branch, (RSma) accessory medial branch, (UD) dorsal branch of the n. ulnaris, (*) communications between the RSm and RSl. White bars: 10 mm.

**Figure 6:**
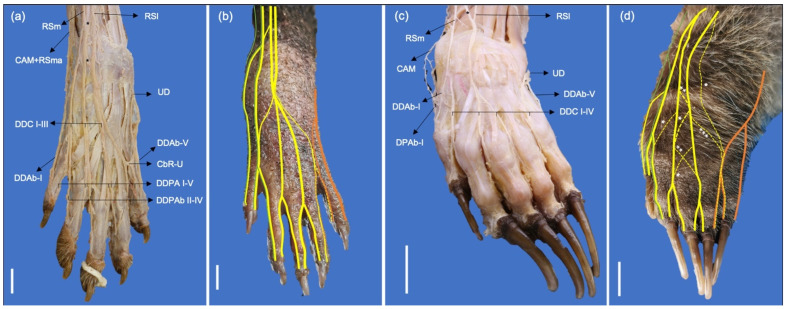
Dorsal photographic views of the dorsal digital nerves: (**a**) Dissection a left manus of a *P. cancrivorus*; (**b**) Scheme of the nerve distribution in a left manus of a *P. cancrivorus*; (**c**) Dissection a left manus of a *N. nasua*; (**d**) Scheme of the nerve distribution in a left manus of a *N. nasua*. (CAM) n. cutaneus antebrachii medialis, (CAM-RSma) common trunk of CAM and RSma, (CbR-U) communicating branches between DDAbIV and DDAV, (DDAb-I) n. digitalis dorsalis I abaxialis, (DDAb-V) n. digitalis dorsalis V abaxialis, (DDAV) n. digitalis dorsalis V axialis, (DDC I-IV) nn. digitales dorsales communes I-IV, (DDPA I-IV) nn. digitales dorsales propii I-IV axiales, (DDPAb II-IV) nn. digitales dorsalis propii II-IV abaxiales, (RS) superficial branch of the n. radialis, (RSl) lateral branch, (RSm) medial branch, (RSma) accessory medial branch, (black asterisks) communications between RSm and RSl, (UD) dorsal branch of the n. ulnaris, (yellow lines) distribution of the superficial branch of the radial nerve, (orange lines) ulnar nerve, (green lines) musculocutaneus nerve, (yellow lines) median nerve. The dashed lines indicate the variant communicating branches. White asterisks indicate the number of times that the communicating branch is present. White bars: 10 mm.

The branches of the superficial branch of the radial nerve (*Ramus superficialis*) are relatively larger in *P. cancrivorus* than in *N. nasua*. In *N. nasua*, it divides into two branches, lateral (*Ramus lateralis*) and medial (*Ramus medialis*), which extend laterally and medially to the cephalic vein, respectively. In *P. cancrivorus*, the superficial branch at the proximal fourth of the antebrachium is a large trunk and distally divides into three branches: lateral, medial, and accessory medial. The accessory medial branch forms at the proximal third of the antebrachium and joins the medial antebrachial cutaneous nerve, and both form the abaxial dorsal digital nerve I and the dorsal common digital nerve I. The accessory medial branch is not present in *N. nasua;* thus, the latter two nerves are formed by the medial branch of the superficial radial nerve. However, there are communicating branches from the medial antebrachial cutaneous nerve at the level of the antebrachium in *N. nasua*. The medial and lateral branches form the common dorsal nerves II and III due to communication between them proximal to the manus and the dorsum of the manus in both species. In *P. cancrivorus*, the lateral branch of the radial nerve and the dorsal branch of the ulnar nerve form the common dorsal digital nerve IV, although the radial nerve contributes mainly to forming the abaxial proper dorsal digital nerve IV, and the ulnar nerve mainly forms the axial proper dorsal digital nerve V. In *N. nasua*, the dorsal branch of the ulnar nerve forms the common dorsal digital nerve IV, although it receives weak communicating branches from the lateral branch of the radial nerve in three cases (Nn2, Nn3, and Nn4). The superficial branch of the radial nerve forms the lateral antebrachial cutaneous nerve (*n. cutaneus antebrachii lateralis*) in *P. cancrivorus*, while it forms from the lateral branch of the superficial branch of the radial nerve in *N. nasua*. However, in one limb of *N. nasua*, it formed similarly to *P. cancrivorus* (Nn4) (Figure 5 and Figure 6).

The musculocutaneous nerve (*N. musculocutaneus*) extends cranially to the axillary and brachial arteries, which form several branches. At the axillary level in *P. cancrivorus*, it forms the branch to the m. coracobrachialis and an ansa axillaris. This latter is named ansa axillaris because it passes medially to the axillary artery and forms two communicating branches, one proximal to the caudal pectoral nerves (*ansa pectoralis*) and one distal to the median nerve (Figure 1). In one specimen bilaterally (Pc4), C7 directly forms the ansa axillaris and not the musculocutaneous nerve. In one specimen unilaterally (Pc3), the ansa axillaris forms a branch to the m. pectorlais profundus. The branch to m. coracobrachialis forms proximally to the ansa axillaris in two specimens (Pc2 and Pc5), while in three specimens, it forms distally. In two specimens bilaterally (Pc1 and Pc2), the ansa pectoralis also communicates with the lateral thoracic nerve (Figure 1 and Figure 3).

**Figure 7:**
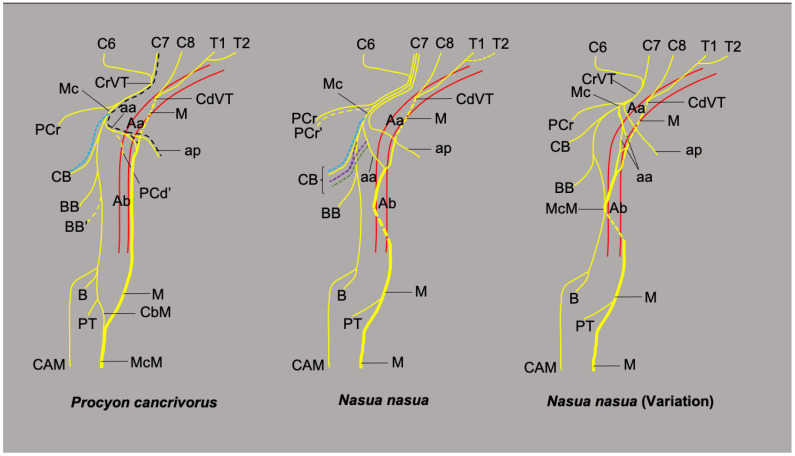
Schemas of the median and musculocutaneous nerves in *P. cancrivorus* and *N. nasua.* (Aa) arteria axillaris, (Ab) arteria brachialis, (aa) ansa axillaris, (ap) ansa pectoralis, (BB) branch to the m. biceps brachii, (BB’) variant branch to BB, (CAM) N. cutaneus antebrachii medialis, (CB) branches to the m. coracobrachialis, (CbM) ramus communicans cum n. mediano, (CdVT) caudal ventral trunk, (CrVT) cranial ventral trunk, (M) n. medianus, (Mc) n. musculocutaneus, (McM) common trunk of Mc and M, (PCd’) variant branch to m. pectoralis profundus, (PCr’) variant origin of the PCr from the cranial ventral trunk, (PT) branch to the m. pronator teres, (black dashed line) anatomical variation where the ansa axillaris is only formed by C7, (blue dashed line) origin of CB proximal to aa, (green dashed line) origin of CB from aa, (purple dashed line) origin of CB from Mc and aa. *Nasua nasua* (Variation): Variation present in one limb (10%) where branches of Mc and M form the ansa axillaris and a common trunk at the level of the brachium.

At the axillary level in *N. nasua*, the ansa axillaris originates from the musculocutaneous nerve and joins the median nerve at the level of the transition between the axillary and brachial arteries (more distal than *P. cancrivorus*). The ansa pectoralis does not form from the musculocutaneous nerve. The origin of the branch to the m. coracobrachialis is variant; therefore, it forms proximally to the ansa axillaris in two limbs (Nn3 bilaterally), from the ansa axillaris in four limbs (two unilaterally—Nn4 and Nn5—and one bilaterally—Nn2), from one branch of the ansa axillaris and another branch proximal to the ansa axillaris in one limb (Nn4), and distally to the ansa axillaris in three limbs (one bilaterally—Nn1—and one unilaterally—Nn5) (Figure 7).

In both species, the proximal muscular branch (*Ramus muscularis proximalis*) of the musculocutaneous nerve forms at the axillary level, and the main trunk of the musculocutaneous nerve continues distally between the brachial artery and the m. biceps brachii. At the distal third of the brachium, the nerve forms three branches: the medial antebrachial cutaneous nerve (*N. cutaneus antebrachii medialis*), the distal muscular branch (*Ramus muscularis distalis*), and a branch to the elbow joint capsule. In *P. cancrivorus*, the main trunk of the nerve continues distally toward the antebrachium, which, at the elbow level, sends branches to the pronator teres and flexor carpi radialis muscles and communicates with the median nerve distally to the supracondylar foramen (*Ramus communicans cum n. mediano*), forming a common trunk toward the manus (Figure 4). In both species, the medial cutaneous antebrachial nerve passes between the brachialis and biceps brachii muscles and extends medially to the m. brachioradialis until the medial side of the manus. Therefore, it sends branches to the medial side of the palmar pad, and in the case of *N. nasua*, it forms the abaxial palmar nerve of digit I. Proximal to the radial styloid process, it sends a communicating branch to the medial branch of the superficial branch of the radial nerve. A communicating branch is also sent to the abaxial dorsal digital nerve I (Figure 7).

On the right brachial plexus of one *N. nasua* (Nn1), the musculocutaneous has a different arrangement at the axillary and brachial regions. The ansa axillaris receives communicating branches from the median nerve, forming an ansa axillaris that is more proximal and medial to the axillary artery. From there, the musculocutaneous and median nerves continue as a common trunk medial to the brachial artery, which receives a communicating branch from the proximal muscular branch of the musculocutaneous nerve. The distal muscular branch and medial antebrachial cutaneous nerve form from a branch of the common trunk at the level of the middle of the brachium (see the right brachial plexus of Figure 2 and the schemas of Figure 7).

**Figure 8:**
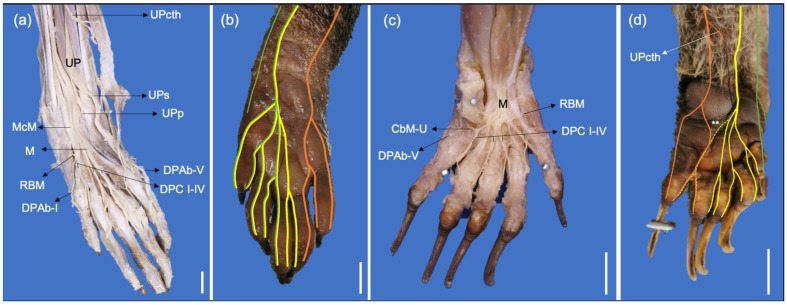
Palmar photographic views of the manus with deep dissections and skin. (**a**) Palmar photographic view of a deep dissection of a right manus of a *P. cancrivorus*; (**b**) Scheme of the palmar nerves in a right manus of a *P. cancrivorus;* (**c**) Palmar photographic view of a deep dissection of a left manus of a *N. nasua*; (**d**) Scheme of the palmar nerves in a left manus of *N. nasua*. (CbM-U) communicating branch from UPs to DPC IV, (DPC I-IV) nn. digitales palmares communes I-IV, (DPAb-I) n. digitalis palmaris I abaxialis, (DPAb-V) n. digitalis palmaris V abaxialis, (M) n. medianus, (MCM) common trunk of median and musculocutaneous nerves, (RBM) recurrent branch of M, (UP) n. ulnaris ramus palmaris, (UPs) ramus superficialis, (UPp) ramus profundus, (UPcth) branch to the distal extreme of the antebrachium and carpal tactile hairs, (yellow lines) distribution of the superficial branch of the radial nerve, (orange lines) ulnar, (green lines) musculocutaneous and (yellow lines) median nerves, (dashed lines) variant communicating branches, (*) number of times that the communicating branch is present. White bars: 10 mm.

The median nerve (*N. medianus*) extends medially to the brachial artery at the proximal half of the brachium and at the distal half lateral to the brachial artery to pass alone through the supracondylar foramen (without vessels). At the level of the antebrachium, the median nerve sends branches to the caudomedial antebrachial muscles but not the flexor carpi ulnaris and capita ulnaris of the flexor digitorum profundus. In *P. cancrivorus*, it does not send branches to m. pronator teres and communicates with the musculocutaneous nerve. The median nerve extends together with the median artery between the flexor carpi radialis and the capita radialis of the m. flexor digitorum profundus to reach the manus passing through the carpal canal. Distal to the carpus, it forms common palmar nerves I to III. It also forms the common palmar nerve IV in one *N. nasua* bilaterally (Nn1), which receives a communicating branch from the superficial palmar branch of the ulnar nerve) (Figure 8).

The ulnar nerve (*N. ulnaris*) extends caudal to the brachial artery and cranial to the brachial vein and is covered by the m. tensor fasciae antebrachii at the level of the brachium. It passes deeply to the m. anconeus medialis just proximally and caudally to the medial epicondyle to continue toward the antebrachium between the m. flexor carpi ulnaris and caput ulnare of the m. flexor digitorum profundus. The nerve divides into dorsal and palmar branches (*Ramus dorsalis* and *Ramus palmaris,* respectively) at the proximal third of the antebrachium in *N. nasua* and at the middle third in *P. cancrivorus*. The dorsal branch sends branches to the distal third of the caudolateral surface of the antebrachium and continues distally to pass superficially between the tendon of the m. flexor carpi ulnaris and the styloid process of the ulna. It passes laterally to the accessory carpal bone toward the dorsum of the manus and forms the common dorsal digital nerve IV and the abaxial dorsal digital nerve V. The palmar branch at the distal fourth of the antebrachium sends a branch to the skin, which extends to the venous sinuses of the tactile carpal hairs and the distal extreme of the antebrachium. This branch passes between the palmaris longus and flexor digitorum superficialis muscles in *N. nasua*, while in *P. cancrivorus,* it perforates the m. palmaris longus. At the carpal level, the palmar branch continues deeply to the flexor retinaculum and medial to the accessory carpal bone, where it forms the superficial and deep branches. The superficial branch forms the abaxial palmar digital nerve V and the common palmar digital nerve IV, and the deep branch forms the metacarpal nerves and innervates most of the intrinsic muscles of the manus (Table 1) (Figure 7 and Figure 8).

The caudal antebrachial cutaneous nerve (*N. cutaneus antebrachii caudalis*) is an independent nerve to the ulnar nerve, which originates directly from T1 or T1-T2. It passes medially to the ulnar nerve at the axillary level, while at the brachial level, it passes medially to the brachial artery and cranially to the m. tensor fasciae antebrachii, reaching the caudal surface of the antebrachium (Figure 1, Figure 2 and Figure 4). The nerve communicates with the branch to the carpal tactile hairs bilaterally in one *N. nasua* (Nn4).

## 4. Discussion

### 4.1. Comparative Origin of the Brachial Plexus in Carnivorans

The origins of the brachial plexus in *P. cancrivorus* and *N. nasua* from C5-T1, C5-T2, and C6-T2 have been found in few carnivorans. C5-T1 origin has only been reported in other caniformes, such as *Meles meles, Nyctereutes procyonoides* [31], and *Canis lupus familiaris* [40], and feliformes, such as *Leopardus geoffroyi* [32], *Puma yagouaroundi* [23], and *Felis catus* [41]. C5-T2 origin has been found in *Potos flavus* [30], *Ailuropoda melanoleuca* [35], *Vulpes vulpes* [22], and *C. l. familiaris* [40]. C6-T2 was reported in *P. flavus* [30], *Bassariscus astutus* [35], *M. meles*, *N. procyonoides, V. vulpes* [31], *C. l. familiaris* [40], and *F. catus* [29]. In contrast, the origin from C6-T1 is the most frequent in most carnivorans (Table 2), which agrees with the brachial plexus evolution in carnivorans, where the ventral branches of C6, C7, C8, and T1 are the largest branches to form the brachial plexus, whereas the branches of C5 and T2 are weak branches [22,30,34,42], as was found in *P. cancrivorus* and *N. nasua*.

Most origins of the brachial plexus nerves in *P. cancrivorus* and *N. nasua* in the present study have also been found in other carnivorans. Some nerve origins of both species have not been found in other carnivorans, although the most of these were found in very low proportions. However, the origin of the median nerve from C6-T1 in *N. nasua* was present in most limbs (60%). The predominant origins of the subscapular, musculocutaneous, axillary, ulnar, long thoracic, lateral thoracic, and cranial pectoral nerves of both procyonids were similar to those of most carnivorans (Table 3). The other nerves had several inter- and intraspecific variations, mainly due to the contribution of C5 and T2 and the presence of the ansa axillaris and ansa pectoralis to the median and caudal pectoral nerves, respectively.

### 4.2. Comparative Distribution of the Brachial Plexus Nerves in Carnivorans and Evolutionary Comments

The brachial plexus nerves conserve a similar distribution among carnivorans mainly to the innervation of the thoracic limb muscles, although some differences have been found. Among them, the suprascapular nerve also innervates other muscles, such as the m. deltoideus in *Martes foina* [43], m. serratus ventralis cervicis in *Puma concolor* [27], and m. trapezius in *F. catus* [28]. One of the subscapular nerves innervates the m. teres major in *M. foina* [43], *A. australis* [26], and *L. geofffroyi* [32]. An independent nerve from the brachial plexus innervates the m. teres major in *P. flavus* [30], *B. astutus,* and *U. americanus* [35], which differs from *P. concolor* [27] and *N. nasua*, where that nerve also innervates the subscapular muscle. The radial nerve also innervates the m. teres minor in *M. foina* [43]. The musculocutaneous nerve may innervate the m. pronator teres as an anatomical variant in *C. thous* [49], which was found in the common pattern of *P. cancrivorus* together with the innervation of the m. flexor carpi radialis. The m. latissimus dorsi may also be innervated by the lateral thoracic nerve in *P. concolor* [27] and *F. catus* [50]. In *M. foina*, the caudal pectoral nerves innervate not only m. pectoralis profundus but also m. cutaneus trunci [43].

Some nerves develop other branches when there are extra muscles. Therefore, the musculocutaneous nerve innervates the caput breve of the m. biceps brachii and the m. coracobrachialis longus in *P. flavus* [30] and *A. melanoleuca* [35]. The median nerve innervates the palmaris longus muscles in *P. flavus* and *P. cancrivorus* [39], as was found in the present study in the latter species and *N. nasua*. The ulnar nerve innervates the m. anconeus medialis (m. anconeus epitrochlearis) in *F. catus* [38], *P. concolor* [27], *A. melanoleuca* [35], *P. flavus* [30], *P. cancrivorus,* and *N. nasua*.

The name n. brachiocephalicus is not in the *NAV* [33], but it has been used by several authors because it innervates m. cleidobrachialis [23,30,31,32,34,45,46], which is a part of m. brachiocephalicus [33]. Other authors named it n. supraclavicularis in *F. catus* [29,51] or n. subclavius in *M. foina* [43] and *Leopardus pardalis* [21]. On the other hand, in *P. concolor*, it was considered a branch of n. suprascapularis to m. cleidobrachialis [27]. In *F. catus*, it was also considered only as a cutaneous branch of the n. suprascapularis, which extends to the cranial skin to the shoulder and brachium [50,52]. In the case of *P. cancrivorus* and a variant manner in *N. nasua,* the latter arrangement was similar since the nerve formed from a common trunk with the n. suprascapularis and did not innervate the m. cleidobrachialis. In another procyonid, *P. flavus*, the brachiocephalic nerve innervated m. cleidobrachialis in only 50% of cases [30]. However, it is mainly innervated by the axillary nerve in all cases of *P. flavus* [30], *N. nasua,* and *P. cancrivorus* [37], which was confirmed in all specimens of the present study. The innervation of m. cleidobrachialis by the axillary nerve has also been described in other caniformes, such as *A. melanoleuca* [35], *A. microtis* [25], and *C. l. familiaris* [42,53], and feliforms, such as *F. catus* [29,50,51] and *P. concolor* [27]. Based on Singh et al. [42], m. cleidobrachialis splits from a primordium different from m. cleidocephalicus in the embryo since the former retains the appropriate innervation by the axillary nerve. Even innervation of m. cleidobrachialis is only reported by the axillary nerve in all domestic mammals [42,53]. Therefore, the present study confirms previous studies [30,37] in which it was concluded that m. cleidobrachialis was evolutionarily derived from m. deltoideus and is its corresponding clavicular part (*Pars clavicularis*). On the other hand, n. brachiocephalicus has developed mainly to innervate the cranial and lateral skin of the shoulder and brachium in carnivorans, which was found in both procyonids.

The communication of the cranial lateral cutaneous brachial nerve of the axillary nerve with the medial branch of the superficial branch of the radial nerve was found in the common pattern of *N. nasua* and *P. cancrivorus*, such as occurs in most carnivorans [24,54,55]. Communication with the lateral branch of the superficial branch of the radial nerve found in lesser proportion in *N. nasua* has been described in *A. melanoleuca* [35]. Therefore, some afferent fibers of the axillary nerve could come from the manus in these species.

The communicating branch from the musculocutaneous nerve to the median nerve in *P. cancrivorus* and *N. nasua* is medial to the axillary artery, as was found in *P. flavus* [30] and other caniformes [35]. Therefore, the communicating branch was called the “ansa axillaris” in the present study, based on the description written by several authors, who have described that this anatomical structure is a communication from the musculocutaneous nerve to the median nerve around the axillary artery [42,44,53,56,57]. The ansa axillaris of *N. nasua* has also been reported previously by other authors in the same species [48,58]. However, it is called a communicating branch of the musculocutaneous nerve with the median nerve at the axillary level [48] or axillary loop [58]. In another study, it was reported as ansa mediana in procyonids, such as *Procyon lotor* and *P. flavus*, while it was absent in the procyonid *B. astutus* [35]. Thus, the ansa axillaris is present in procyonids of the genera *Procyon*, *Nasua,* and *Potos*. However, in the case of *P. cancrivorus*, the ansa axillaris is formed more proximally than in other procyonid species. Other names were given to the ansa axillaris, such as axillary loop by Arlamowska-Palider [58], ansa mediana by Davis [35], and communicating branch at the axillary level by Grzeczka and Zdun [31]. Therefore, the ansa axillaris is also present in mustelids such as *Neovison vison* [31], *M. martes* [31,58], *M. foina* [58], *M. meles* [31,58], *Mustela lutreola*, *Mustela putorius*, and *Mustela nivalis* [58]; ursids such as *Ailuropoda melanoleuca*, *Ursus americanus* [35], and *Ursus arctos* [58]; the ailurid *Ailurus fulgens* [35]; and the canid *N. procyonoides* [31]. In the case of *M. meles*, the ansa axillaris is formed by two branches [31], but both communicate with the median nerve, while in *P. cancrivorus,* one of the branches communicates with the caudal pectoral nerves. Therefore, the presence of an ansa axillaris in arctoid carnivorans (infraorder Arctoidea, where the Musteloidea and Ursoidea superfamilies are included [1,4]) can be normal and differs from other authors who described that carnivorans do not have ansa axillaris [44]. Even its presence in a canid such as *N. procyonoides* [31] allows us to suggest that the presence of an ansa axillaris in carnivorans is not only due to its phylogenetic relationship from the last common ancestor of Caniformes, but also due to the anatomical shape of its body. *N. procyonoides* has a body shape very similar to procyonids of the genus *Procyon* but is phylogenetically a canid [1,4]. However, it may be absent as an anatomical variant in *N. procyonoides* since it was not reported in one specimen dissected by Davis [35], though the absence is the common pattern to most canids [31,34,35,45,46,58]. Therefore, when an ansa axillaris is present in some canids different from *N. procyonoides,* it could be considered an aberrant arrangement of the n. musculocutaneous since, as was reported in one *C. l. familiaris,* the formation of an ansa axillaris could obstruct the blood flow of the axillary artery [56].

A common trunk of the musculocutaneous and median nerves at the brachial level has been found in Mephitis and some specimens of M. putorius [58], similar to that which occurred in one case of *N. nasua*. This common trunk of both nerves is a normal arrangement in ungulates [44,53,58] and pangolins [59]. Therefore, it is a primitive arrangement that could have been present in the last common ancestor of Carnivora, Ungulata, and Pholidota [60]. Consequently, the common trunk of both nerves may be present as an anatomical variant in some carnivorans.

The communicating branch of the musculocutaneous nerve with the median nerve at the elbow level was absent in all cases of *N. nasua*, which confirms other studies performed in the same species [48,58]. In another procyonid, *P. flavus*, it may be present but as a weak branch [30]. In *P. cancrivorus,* it is a large branch that is more a continuation of the musculocutaneous nerve together with the median nerve than a communicating branch. In one case of *C. l. familiaris,* it was reported that the musculocutaneous nerve did not join the median nerve but did not continue distally at the antebrachial region, replacing the median nerve to form common digital nerves I to III [58]. In other carnivorans, the unique communication at the elbow level is normal, as has been reported in canids [31,34,45,46,49,58], an otariid such as *A. australis* [26], and felids [23,24,32]. However, this communicating branch may be absent as a variation in felids [27,28,29,58]. Therefore, the main nerve of the brachial plexus that presents changes in carnivorans is the musculocutaneous nerve, mainly in the presence of communicating branches with the median nerve. In the case of *P. cancrivorus*, the musculocutaneous nerve was a large nerve with two communicating branches (*ansa axillaris* and *ramus comunicans*), which differs from most carnivorans, who only have one communicating branch.

The median nerve passes through the supracondylar foramen within vessels in *A. melanoleuca* [35], *P. cancrivorus,* and *N. nasua*, while it passes with the brachial artery in mustelids [31] and F. catus [42,61] and even with the brachial vein in *P. flavus* [30], *P. concolor* [24,27], and *P. onca* [24]. In *A. melanoleuca*, it has been reported that the median nerve also innervates the m. flexor carpi ulnaris [35]. However, when we observe the figures of the antebrachial muscles of *A. melanoleuca* presented by Davis [35], the caput humerale of the m. flexor carpi ulnaris he identified could actually be the palmaris longus lateralis found in *P. flavus* [39] or the palmaris longus of *P. cancrivorus* and *N. nasua*. It has a similar location in procyonids and is innervated by the median nerve. Therefore, palmaris longus or palmaris longus lateralis could have evolutionarily derived from m. flexor digitorum superficialis, not only from the last common ancestor of musteloids but also within arctoid carnivorans (based on the phylogenetic three of carnivorans [1]).

The ulnar nerve always innervates the m. anconeus medialis in both procyonids, similar to other carnivorans that present it [27,30,49,51,62]. This corroborates the fact that the muscle was evolutionarily derived from the m. flexor carpi ulnaris, as has been found in most mammals [63]. It may only be derived from the triceps brachii and flexor carpi ulnaris muscles in other phylogenetically distant mammals, such as xenarthrans, since in some of them, it is innervated by the radial and ulnar nerves [64]. Thus, it is not a head derived from the m. triceps brachii in carnivorans.

The branch from the ulnar nerve to innervate the carpal tactile hairs has only been reported in *F. catus* [29]. In *N. nasua,* the carpal tactile hairs were highly developed, while in *P. cancrivorus*, they were very small and short. The carpal tactile hairs in *N. nasua* should help to locate prey underground while digging, since these hairs may collect information about substrate vibration [65,66]. The carpal tactile hairs also collect information about surface properties related to substrate diameter, which is necessary to influence the body and thoracic limb postures while walking in several substrates [65]. However, the homologous branch from the ulnar nerve to the carpal tactile hairs is present in both species, similar to that reported in *P. flavus* [39]. Thus, although the carpal tactile hairs are more developed in *Nasua*, the distal fourth of the antebrachium remains innervated by the ulnar nerve in procyonids.

The caudal antebrachial cutaneous nerve originated independently from T1 or T1-T2, such as in *P. cancrivorus* and *N. nasua*, and has also been described in *P. flavus* [30], *F. catus* [29,51], and *A. melanoleuca* [35], while in other carnivoran species, it is a branch of the ulnar nerve. However, in a study that stimulated the dorsal roots of C5 to T2 in *C. l. familiaris,* the caudal antebrachial cutaneous nerve originated predominantly from T1 and T2 [67]. Therefore, the caudal surface of the antebrachium is predominantly innervated by the thoracic spinal nerves in these species of carnivorans.

The radial nerve innervated the m. brachialis in two cases of *P. cancrivorus*, similar to *A. melanoleuca* [35]; however, its main innervation is from the musculocutaneous nerve. Therefore, in these cases, the m. brachialis could embryologically have derived not only from the myotomes that migrate with the musculocutaneous nerve but also from the radial nerve. This could be a primitive phylogenetic arrangement within a last common ancestor of reptiles and mammals since, in some reptiles, a part of the radial nerve goes within a common trunk with the musculocutaneous nerve [68,69]. The branches to the craniolateral antebrachial muscles in carnivorans seem consistent [68,70,71,72], although the course of the deep branch of the radial nerve with respect to the m. supinator is variable. It passes superficially to the m. supinator in *P. flavus* [30] and *F. catus* [29,51] and passes deeply to the m. supinator in *C. l. familiaris* [55], *A. melanoleuca* [35], *P. cancrivorus,* and *N. nasua.*

The presence of an ansa pectoralis in *P. cancrivorus* and *N. nasua* has also been found in other procyonids, such as *P. flavus* [30] and *B. astutus* [35], and even in other arctoids, such as *A. fulgens*, *A. melanoleuca,* and *U. americanus* [35]. It could be a primitive arrangement due to the pectoralis superficialis and profundus muscles of mammals evolutionarily derived from an undivided pectoral muscle of reptiles [73]. Thus, the pectoral nerve to the reptilian pectoral muscle was divided into cranial and caudal pectoral nerves during the transition to mammals when the muscle divided into superficial and deep pectoral muscles; thus, communicating branches between both nerves may be present in carnivorans. The innervation of the m. pectoralis transversus from the caudal pectoral nerves has only been found in procyonids [30,37], which was further strengthened by findings in the present study with more specimens. This indicates that the m. pectoralis transversus in procyonids was derived not only from the m. pectoralis superficialis but also from the m. pectoralis profundus of the last common ancestor of procyonids [37].

The lateral thoracic nerve innervates m. cutaneus trunci in all carnivorans, although in some carnivorans, it also innervates m. pectoralis profundus, such as in *M. martes*, *V. vulpes* [31], *C. thous* [45], *L. gymnocercus* [46], *F. catus* [28], *P. yagouaroundi* [23], and *L. geoffroyi* [32]. In *P. cancrivorus* and *N. nasua*, it innervates the m. pectoralis abdominalis, which has been considered a part of the m. pectoralis profundus by some authors. However, in a recent study, it was hypothesized that the m. pectoralis abdominalis of carnivorans is an evolutionary derivation from m. cutaneus trunci of the last common ancestor of placental and marsupial mammals, but not from m. pectoralis profundus [37]. Even in a monotreme such as the platypus (*Ornithorhynchus anatinus*), both nerves also originate from a common trunk [74]. Therefore, the common trunk of the caudal pectoral and lateral thoracic nerves supports the hypothesis that the m. cutaneus trunci of mammals is derived from the pectoralis muscle of reptiles [73] and the m. pectoralis abdominalis from the m. cutaneus trunci in carnivorans [37].

### 4.3. Comparative Nerve Branching on the Manus of Carnivorans

The branching of the medial and lateral branches of the superficial radial nerve at the dorsum of the manus in *P. cancrivorus* and *N. nasua* is highly variable due to several communications between them. However, the communications between them in *P. cancrivorus* are by large branches of almost the same thickness as the main branch. In other carnivorans, both branches have a common pattern. Therefore, the medial branch forms the abaxial dorsal digital nerve I and the common dorsal digital nerve I, and the lateral branch forms the common dorsal digital nerves II to IV in canids, mustelids, and ursids [31,34,35,43,54]. In *P. cancrivorus* and *N.* nasua, the common dorsal digital nerves II and III originate from contributions of the lateral and medial branches of the superficial radial nerve. The medial cutaneous antebrachial nerve communicates with the medial branch of the radial nerve in felids [24,54] and procyonids, such as *N. narica* [54], *N. nasua,* and *P. cancrivorus*. Therefore, the axial and abaxial dorsal surfaces of digit I are innervated by the musculocutaneous and radial nerves in these species.

In *P. cancrivorus*, the most lateral branch forms the abaxial dorsal digital nerve IV, and this branch communicates with the axial dorsal digital nerve V, having a similar arrangement to that found in *P. flavus* [30] and *A. melanoleuca* [35]. In a feliform such as *F. catus*, the lateral branch only forms the dorsal common digital nerves II to III, while the dorsal branch of the ulnar nerve forms the dorsal common digital nerve IV [28,29,52]. However, a communicating branch from the common dorsal digital nerve III (radial nerve) to the common dorsal digital nerve IV (ulnar nerve) has been found in felids [29,54]. Similarly, it also occurs in mustelids [43,54], ursids, and procyonids such as *N. narica* [54] and *N. nasua.* In *P. onca*, the radial nerve does not innervate digit IV and is, thus, supplied axially and abaxially by the dorsal branch of the ulnar nerve [24]. In *P. concolor*, dorsal common digital nerve III may be formed by communication between the lateral branch of the radial nerve and the dorsal branch of the ulnar nerve [54].

The medial cutaneous antebrachial nerve forms the abaxial palmar digital nerve I in *P. flavus* [30], similar to that found in *N. nasua*. The abaxial palmar digital nerve I in *P. cancrivorus* is formed by the median nerve, which occurs normally in other carnivorans [24,28,29,31,34,43].

The ulnar nerve forms the palmar common digital nerve IV in canids [31,34], mustelids [31,43], procyonids such as *P. cancrivorus* and *N. nasua*, and a felid such as *F. catus* [24,28,29]. It is formed by the median nerve in a ursid as *A. melanoleuca* [35], in a procyonid as *P. flavus* [30], and as an anatomical variant in *N. nasua*. However, in *A. melanoleuca* [35] and *N. nasua*, it may receive a communicating branch from the ulnar nerve. In felids such as *P. concolor* and *P. onca*, the median nerve does not innervate digit IV since the axial and abaxial palmar surfaces are innervated by the ulnar nerve [24].

The branches of the ulnar and median nerves to the intrinsic muscles of the manus in *N. nasua* and *P. cancrivorus* were similar to those reported in *P. flavus* [30], *C. l. familiaris* [34], and felids [24]. However, the innervation by the median nerve to the most medial lumbrical muscle in both procyonids was not described in those species, although in *C. l. familiaris*, it is normal because the fourth lumbrical muscle is absent [55].

The large size of the palmar branches to the digits from the median, musculocutaneous, and ulnar nerves together with the large dorsal branches of the superficial branch of the radial nerve in *P. cancrivorus* corroborates the anatomical adaptation of the nervous peripheral system of the genus *Procyon* to allow a higher touch sensitivity than other carnivorans. Even in *P. lotor*, a high innervation has been found with the presence of all types of sensory receptors in similar proportions compared with other mammals, such as primates [75,76].

## 5. Conclusions

The brachial plexus of both procyonid species originates in a variant manner from C5-T1, C5-T2, C6-T1, or C6-T2, from which form the thoracic limb nerves having a similar distribution to the muscles and skin. The major nerves, such as the radial and median nerves, originated from more spinal nerves than other nerves in both procyonids, which could be an anatomical adaptation to increase the function of the antebrachial muscles and skin sensitivity of the antebrachium and manus. However, some specific differences could be associated with the locomotor behavior in each species. The higher contribution from C5 in *N. nasua* to the suprascapular and cranial subscapular nerves could be associated with a higher use of the scapular muscles to move the shoulder during digging. In addition, the innervation from the ulnar nerve to the sinusoids of the carpal tactile hairs should help to feel invertebrates underground and coordinate the movements in several terrestrial and arboreal substrates. On the other hand, *P. cancrivorus* has a higher contribution from T2, two communications from the musculocutaneous nerve to the median nerve, and relatively larger nervous branches to the skin. This arrangement could indicate that the thoracic limb nerves in *P. cancrivorus* have been more adapted to touch and greater manual skills than those in *N. nasua,* since *P. cancrivorus* uses its manus to bring the food to the mouth and even to search for food at night and underwater.

In general, through a comparative study with other species, the distribution and branching of the brachial plexus nerves also depends on the evolutive derivation of the muscles. The main nerve of the brachial plexus that presents changes in carnivorans is the musculocutaneous nerve, mainly due to the presence of communicating branches with the median nerve. The presence of ansa axillaris and ansa pectoralis in both procyonids mainly has a phylogenetic relationship within the infraorder Arctoidea since both structures can be in ursoid and musteloid species. The communicating branches between nerves also have a phylogenetic relationship not only with the common ancestor of carnivorans but also with other mammals and even reptiles. The dorsal sensory innervation of the manus is highly variable in most carnivorans due to the dorsal communicating branches and does not depend on the taxonomic family. However, in canids, it seems constant.

## Figures and Tables

**Table 1:** Origin and distribution of the brachial plexus nerves in *Procyon cancrivorus* and *Nasua nasua*.

Nerve	Origin	*Procyon cancrivorus* (%)	*Nasua nasua* (%)	Muscle Innervation	Skin Innervation
*Suprascapularis*	C5-C6	10	80	Supraspinatus and infraspinatus	
	C5-C7	10	0		
	C6	70	10		
	C6-C7	10	10		
*Subscapularis cranialis*	C5-C6	0	60	Subscapularis	
	C6	70	30		
	C6-C7	30	10		
*Subscapularis medium*	absent	90	20		
	C6	10	30		
	C6-C7	0	50		
*Subscapularis caudalis*	C6-C7	100	100		
*Teres major*	C6-C7	20	100	Teres major and subscapularis	
*Axillaris*	C6-C7	100	100	Subscapularis, teres, minor, deltoideus (Pars scapularis, pars acromialis, pars clavicularis -m. Cleidobrachialis-), teres major (only in *P. cancrivorus*).	Cranial, lateral, and medial surfaces of the distal half of the brachium and proximal extreme of the antebrachium.
*Musculocutaneus*	C6-C7	90	100	Coracobrachialis, biceps brachii, brachialis, pronator teres (only in *P. cancrivorus*), and flexor carpi radialis (only in *P. cancrivorus*).	Medial surfaces of the antebrachium and manus. Abaxial and axial dorsal surfaces of the digit I, and abaxial dorsal surface of the digit II (via communicating branches with the radial nerve). Abaxial and axial palmar surfaces of the digits I and II, and abaxial palmar surface of the digit III (via common trunk with the median nerve in *P. cancrivorus*) Abaxial palmar surface of the digit I (*N. nasua*)
	C7	10	0		
*Radialis*	C6-C8	20	0	Tensor fasciae antebrachii, triceps brachii (Caput longum, caput mediale, caput accessorium, and caput laterale), anconeus lateralis (anconeus), brachioradialis, extensor carpi radialis, extensor digitorum communis, extensor digitorum lateralis, extensor carpi ulnaris, supinator, abductor digiti I longus, and extensor digiti I et II.	Cranial and lateral surfaces of the antebrachium, and dorsal and medial surfaces of the manus Axial and abaxial dorsal surfaces of the digits I to III. Abaxial dorsal surface of the digit IV and axial dorsal surface of the digit V (100% in *P. cancrivorus* and 40% in *N. nasua*)
	C6-T1	20	60		
	C6-T2	60	0		
	C7-T1	0	40		
*Medianus*	C6-T1	20	60	Pronator teres (only in *N. nasua*), flexor carpi radialis, flexor digitorum superficialis, palmaris longus, flexor digitorum profundus (caput humerale and caput radiale), interflexorii, pronator quadratus, abductor digiti I brevis, flexor digiti I brevis, and the most medial lumbricalis.	Axial and abaxial palmar surfaces of the digits II to III. Abaxial palmar surface of the digit I (*P. cancrivorus*) Abaxial palmar surface of the digit IV and axial palmar surface of the digit V (10% of *N. nasua*)
	C6-T2	50	40		
	C7-T2	30	0		
*Ulnaris*	C8-T1	20	60	Anconeus medialis (Anconeus epitrochlearis), flexor carpi ulnaris (Caput humerale and caput ulnare), flexor digitorum profundus (Caput humerale and caput ulnare), flexor digitorum brevis, adductor digiti I, adductor digiti II, abductor digiti V, flexor digiti V, adductor digiti V, interossei, and the three lateral lumbricales.	Caudal surface of the antebrachium. Carpal tactile hairs and distal extreme of the antebrachium. Lateral dorsal and palmar surfaces of the manus. Axial and abaxial palmar surfaces of the digit V. Abaxial palmar and dorsal surfaces of the digit IV. Axial and abaxial dorsal surfaces of the digit V.
	C8-T2	80	40		
*Cutaneus antebrachii caudalis*	C8-T1	0	10		Caudal surface of the antebrachium
	C8-T2	60	20		
	T1	20	70		
	T1-T2	20	0		
*Brachiocephalicus*	C5-C6	60	80	Supraspinatus (only 10% in *P. cancrivorus*)	Cranial surface of the shoulder and proximal half of the cranial surface of the brachium
	C6	30	20		
	C6-C7	10	0		
*Thoracicus longus*	C7	100	90	Serratus ventralis thoracis	
	C7-C8	0	10		
*Thoracodorsalis*	C6-C8	20	10	Latissimus dorsi	
	C6-T1	20	10		
	C6-T2	20	0		
	C7-C8	20	80		
	C7-T2	20	0		
*Pectorales craniales*	C6-C7	90	40	Pectoralis descendens and pectoralis transversus	
	C7	0	60		
	C7-T2	10	0		
*Pectorales caudales*	C6-T1	20	20	Pectoralis profundus and pectoralis transversus	
	C6-T2	50	0		
	C7-T1	0	80		
	C7-T2	30	0		
*Thoracicus lateralis*	C6-T2	40	0	Cutaneus trunci and pectoralis abdominalis	
	C7-T1	0	10		
	C8-T1	20	90		
	C8-T2	40	0		

**Table 2:** Comparative origins of the brachial plexus in carnivorans.

Suborder	Infraorder	Superfamily	Family	Species	Number of Limbs (Number of Specimens)	Brachial Plexus Origin (%)
Caniformia	Arctoidea	Musteloidea	*Procyonidae*	*Procyon cancrivorus*	10 (2 females and 3 males)	C5-T1 (20%) C6-T2 (80%)
				*Nasua nasua*	10 (1 female and 4 males)	C5-T1 (20%) C5-T2 (60%) C6-T1 (20%)
				*Bassariscus astutus* [35]	2 (1 unreported sex)	C6-T2 (100%)
				*Potos flavus* [30]	10 (3 females and 2 males)	C5-T2 (30%) C6-T2 (70%)
			*Mustelidae*			
				*Martes foina* [43]	12 (3 females and 3 males)	C6-T1 (100%)
				*Martes martes* [31]	16 (6 female and 2 male)	C6-T1 (100%)
				*Neovison vison* [31]	60 (18 female and 12 male)	C6-T1 (26.67%) C6-T2 (73.33%)
				*Neovison vison* [44]	52 (26 unreported sexes)	C6-T1 (100%)
				*Meles meles* [31]	50 (15 females and 10 males)	C5-T1 (10%) C6-T1 (40%) C6-T2 (50%)
		Ursoidea	*Ursidae*	*Ailuropoda melanoleuca* [35]	2 (1 male)	C5-T2 (100%)
				*Ursus americanus* [35]	2 (1 unreported sex)	C6-T1 (100%)
		Canoidea	*Canidae*	*Nyctereutes procyonoides* [31]	40 (10 female and 10 males)	C5-T1 (12.5%) C6-T1 (25%) C6-T2 (62.5%)
				*Vulpes vulpes* [31]	52 (10 females and 16 male)	C5-T1 (7.69%) C6-T1 (61.54%) C6-T2 (30.77%)
				*Vulpes vulpes* [22]	12 (6 males)	C5-T2 (33.33%) C6-T1 (66.67%)
				*Canis lupus familiaris* [40]	58 (unreported specimens and sexes)	C6-T1 (58.62%) C6-T1 (20.69%) C5-T1 (17.24%) C5-T2 (0.034%)
				*Atelocynus microtis* [25]	1 female	C6-T1 (100%)
				*Cerdocyon thous* [45]	20 (8 females and 2 males)	C6-T1 (100%)
				*Cerdocyon thous* [20]	6 (3 males)	C6-T1 (100%)
				*Lycalopex gymnocercus* [46]	20 (3 females and 7 males)	C6-T1 (100%)
		Pinnipedia	*Otaridae*	*Arctocephalus australis* [26]	4 (1 female and 1 male)	C6-T1 (100%)
Feliformia	Aeluroidea	Feloidea	*Felidae*	*Felis catus* [47]	12 (6 unreported sexes)	C6-T1 (100%)
				*Felis catus* [28]	10 (5 females)	C6-T1 (100%)
				*Panthera onca* [47]	4 (2 unreported sexes)	C6-T1 (100%)
				*Puma concolor* [47]	6 (3 unreported sexes)	C6-T1 (100%)
				*Puma concolor* [27]	2 (1 female)	C6-T1 (100%)
				*Puma yagouaroundi* [23]	14 (5 males and 2 females)	C5-T1 (57%)
						C6-T1 (43%)
				*Leopardus geoffroyi* [32]	6 (4 females)	C5-T1 (66.66%) C6-T1 (33.33%)
				*Leopardus pardalis* [21]	4 (1 male and 1 female)	C6-T1 (100%)

**Table 3:** Comparative origins of the brachial plexus nerves in carnivorans.

Nerves	Origin	Species
*Suprascapularis*	C5-C6	*P. cancrivorus* (10%), *N. nasua* (80%), *P. flavus* (20%), *L. geofffroyi* (50%), and *C. l. familiaris.*
	C5-C7	*P. cancrivorus* (10%), *P. flavus* (10%), V. vulpes* (33.33%), *M. meles, N. procyonoides, V. vulpes,* and *C. l. familiaris.*
	C6	*P. cancrivorus* (70%), *N. nasua* (10%), *C. thous* (25%), *L. gymnocercus* (20%), C. l. familiaris, F. catus* (80%), P. concolor*, P. yagouaroundi (85.7%), and *L. geofffroyi* (50%).
	C6-C7	*P. cancrivorus* (10%), *N. nasua* (10%), *P. flavus* (70%), *M. meles, M. foina*, *M. martes*, *N. vison, N. procyonoides, C. l. familiaris, A. microtis*, *C. thous* (75%), C. thous*, L. gymnocercus (75%), A. australis, V. vulpes* (33.33%), V. vulpes, F. catus* (20%), *P. yagouaroundi* (14.3%), and *L. pardalis* (100%).
*Subscapulares*	C5-C7	*V. vulpes* (16.67%) and *N. nasua* (60%).
	C6-C7	*P. cancrivorus* (100%), *N. nasua* (40%), *P. flavus* (100%), *N. vison, M. meles, C. l. familiaris, C. thous* (45%), C. thous*, L. gymnocercus (90%), V. vulpes* (83.33%), *V. vulpes, P. yagouaroundi* (78.7%), *L. geofffroyi* (100%), P. concolor*, and *L. pardalis* (100%).
*Musculocutaneus*	C6-C7	*P. cancrivorus* (90%), *N. nasua* (100%), *P. flavus* (100%), *N. vison, M. martes, M. meles, V. vulpes, N. procyonoides, C. l. familiaris, A. microtis, C. thous* (30%), C. thous*, L. gymnocercus (20%), F. catus* (100%), *F. catus, P. onca, P. concolor*, P. concolor*, *P. yagouaroundi* (57.3%), *L. geofffroyi* (50%), and *L. pardalis* (100%).
	C7	*P. cancrivorus* (10%), N. nasua* (100%).
*Axillaris*	C6-C7	*P. cancrivorus* (100%), *N. nasua* (100%), *P. flavus* (80%), *A. microtis, C. thous* (20%), V. vulpes, F. catus* (100%), *F. catus, P. onca, P concolor*, P concolor*, *P. yagouaroundi* (57.3%), and *L. geofffroyi* (50%)
*Radialis*	C6-C8	*P. cancrivorus* (20%) and *C. thous* (5%).
	C6-T1	*P. cancrivorus* (20%), *N. nasua* (60%), and *P. yagouaroundi* (14.3%).
	C6-T2	*P. cancrivorus* (60%), and *P. flavus* (90%).
	C7-T1	*N. nasua* (40%), *N. vison, M. martes*, *M. meles, C. l. familiaris, C. thous* (70%), C. thous*, L. gymnocercus (70%), V. vulpes, N. procyonoides, F. catus, P. onca, P. concolor, P. concolor*, L. geoffroyi (66.67%), and *P. yagouaroundi* (64.3%), and *L. pardalis* (100%).
*Medianus*	C6-T1	*N. nasua* (60%) and *P. cancrivorus* (20%).
	C6-T2	*P. cancrivorus* (50%), *N. nasua* (40%), and *P. flavus* (100%).
	C7-T2	*P. cancrivorus* (30%), *N. vison,* and *C. l. familiaris.*
*Ulnaris*	C8-T1	*P. cancrivorus* (20%), *N. nasua* (60%), *N. vison, M. meles, M. martes*, *N. procyonoides, C. l. familiaris, A. microtis, C. thous* (80%), C. thous*, L. gymnocercus (70%), V. vulpes, A. australis, F. catus* (100%), *F. catus, P. onca, P concolor, P. yagouaroundi* (85.7%), *L. geoffroyi* (100%), and *L. pardalis* (100%).
	C8-T2	*P. cancrivorus* (80%), *N. nasua* (40%), *P. flavus* (100%), *N. vison, M. meles, N. procyonoides,* and *C. l. familiaris.*
*Brachiocephalicus*	C5-C6	*P. cancrivorus* (60%), *N. nasua* (80%), *P. flavus* (30%), *M. meles, N. procyonoides,* and *C. l. familiaris.*
	C5-C7	*P. cancrivorus* (10%).
	C6	*P. cancrivorus* (30%), *N. nasua* (20%), *P. flavus* (60%), *N. vison, M. martes*, *M. foina*, *C. l. familiaris, C. thous* (100%), *L. gymnocercus* (100%), *V. vulpes, P. yagouaroundi* (29.6%), and *L. geoffroyi* (33.34%).
	C6-C7	*P. cancrivorus* (10%) and *L. pardalis* (100%).
*Thoracicus longus*	C7	*P. cancrivorus* (100%), *N. nasua* (90%), *P. flavus* (100%), *M. meles*, *C. l. familiaris, C. thous* (100%), *L. gymnocercus* (100%), V. vulpes, F. catus* (100%), P. concolor*, P. yagouaroundi (92.9%), and *L. geoffroyi* (100%).
	C7-C8	*N. nasua* (10%), *N. vison, M. martes*, *A. australis,* and V. vulpes* (83.33%).
*Thoracodorsalis*	C6-C8	*P. cancrivorus* (20%), *N. nasua* (10%), and *P. flavus* (10%).
	C6-T1	*P. cancrivorus* (20%) and *N. nasua* (10%).
	C6-T2	*P. cancrivorus* (20%) and *P. flavus* (60%).
	C7-C8	*P. cancrivorus* (20%), *N. nasua* (80%), *M. foina*, *M. martes*, *M. meles, C. l. familiaris, C. thous* (5%), *L. gymnocercus* (50%), *V. vulpes, F. catus* *(80%), P. concolor*, P. yagouaroundi (7.1%), and *L. geoffroyi* (83.33%).
*Thoracicus lateralis*	C6-T2	*P. cancrivorus* (40%) and *P. flavus* (10%).
	C7-T1	*N. nasua* (10%), *C. thous* (25%), *L. gymnocercus* (35%), and *P. yagouaroundi* (14.2%).
	C8-T1	*P. cancrivorus* (20%), *N. nasua* (90%), *M. martes*, *N. procyonoides, C. l. familiaris, C. thous* (40%), C. thous*, L. gymnocercus (45%), F. catus* (100%), *P. yagouaroundi* (50%), and *L. geoffroyi* (16.67%).
	C8-T2	*P. cancrivorus* (40%), *P. flavus* (90%), *N. vison, N. procyonoides.*
*Pectorales craniales*	C6-C7	*P. cancrivorus* (90%), *N. nasua* (40%), *P. flavus* (60%), *C. thous* (25%), *L. gymnocercus* (5%), V. vulpes* (33.33%), *V. vulpes, P. yagouaroundi* (21.4%), and *L. geoffroyi* (16.67%).
	C7	*N. nasua* (60%), *P. flavus* (10%), *N. vison, M. meles, M. martes*, *M. foina*, *N. procyonoides, C. thous* (25%), *L. gymnocercus* (40%), F. catus* (100%), P. concolor*, P. yagouaroundi (35.7%), and *L. geoffroyi* (50%).
	C7-T2	*P. cancrivorus* (10%).
*Pectorales caudales*	C6-T1	*P. cancrivorus* (10%) and *N. nasua* (10%).
	C6-T2	*P. cancrivorus* (50%) and *P. flavus* (20%).
	C7-T1	*N. nasua* (80%), *M. martes*, *N. vison, C. thous* (20%), and *L. gymnocercus* (35%).
	C7-T2	*P. cancrivorus* (30%).

References: *Procyonidae: P. cancrivorus* (present study)*, N. nasua* (present study), N. nasua* [48], and *P. flavus* [30]. *Mustelidae: M. foina* [43], *M. martes*, *N. vison,* and *M. meles* [31]. *Canidae*: *N. procyonoides, V. vulpes* [31], V. vulpes* [22], *C. l. familiaris* [34], *A. microtis* [25], *C. thous* [45], C. thous* [20], and *L. gymnocercus* [46]. *Otaridae*: *A. australis* [26]. *Felidae: F. catus, P. onca, P. concolor* [24,47], F. catus* [28], P. concolor* [27], *P. yagouaroundi* [23], *L. geoffroyi* [32], and *L. pardalis* [21].

## Data Availability

The data presented in this study are available on request from the corresponding author. The data are not publicly available due to the high quantity of photos.

## Appendix A

**Table A1:** Origins of the brachial plexuses in five specimens of *P. cancrivorus*.

Nerve	Pc1 (JF)		Pc2 (JF)		Pc3 (AM)		Pc4 (AM)		Pc5 (AM)	
	Right	Left	Right	Left	Right	Left	Right	Left	Right	Left
Brachial plexus	C6-T2	C6-T2	C6-T2	C6-T2	C6-T2	C6-T2	C6-T2	C6-T2	C5-T1	C5- T1
*Suprascapularis*	C6	C6	C6-C7	C6	C6	C6	C6	C6	C5-C7	C5-C6
*Subscapularis cranialis*	C6	C6	C6-C7	C6	C6	C6	C6	C6	C6-C7	C6-C7
*Subscapularis medium*	---	---	---	---	---	---	---	C6	---	---
*Subscapularis caudalis*	C6-C7	C6-C7	C6-C7	C6-C7	C6-C7	C6-C7	C6-C7	C6-C7	C6-C7	C6-C7
*Musculocutaneus*	C6-C7	C6-C7	C7	C6-C7	C6-C7	C6-C7	C6-C7	C6-C7	C6-C7	C6-C7
*Axilaris*	C6-C7	C6-C7	C6-C7	C6-C7	C6-C7	C6-C7	C6-C7	C6-C7	C6-C7	C6-C7
*Radialis*	C6-T2	C6-T2	C6-T2	C6-T2	C6-C8	C6-C8	C6-T2	C6-T2	C6-T1	C6-T1
*Medianus*	C6-T2	C6-T2	C7-T2	C6-T2	C6-T2	C6-T2	C7-T2	C7-T2	C6-T1	C6-T1
*Ulnaris*	C8-T2	C8-T2	C8-T2	C8-T2	C8-T2	C8-T2	C8-T2	C8-T2	C8-T1	C8-T1
*Cutaneus antebrachii caudalis*	C8-T2	C8-T2	C8-T2	C8-T2	C8-T2	C8-T2	T1-T2	T1-T2	T1	T1
*Brachiocephalicus*	C6	C6	C6-C7	C6	C5-C6	C5-C6	C5-C6	C5-C6	C5-C6	C5-C6
*Thoracicus longus*	C7	C7	C7	C7	C7	C7	C7	C7	C7	C7
*Thoracicus lateralis*	C6- T2	C6- T2	C6-T2	C6- T2	C8-T2	C8-T2	C8-T2	C8-T2	C8-T1	C8-T1
*Thoracodorsalis*	C7-T2	C7-T2	C7-C8	C7-C8	C6-C8	C6-C8	C6-T2	C6-T2	C6-T1	C6-T1
*Pectorales craniales*	C6-C7	C6-C7	C7-T2	C6-C7	C6-C7	C6-C7	C6-C7	C6-C7	C6-C7	C6-C7
*Pectorales caudales*	C6-T2	C6-T2	C7-T2	C6-T2	C6-T2	C6-T2	C7-T2	C7-T2	C6-T1	C6-T1

AM: adult male, JF: juvenile female, Pc#: number of the specimen, ---: absent.

**Table A2:** Origins of the brachial plexuses in five specimens of *N. nasua*.

Nerve	Nn1 (IM)		Nn2 (JM)		Nn3 (AF)		Nn4 (AM)		Nn5 (AM)	
	Right	Left	Right	Left	Right	Left	Right	Left	Right	Left
Brachial plexus	C5-T2	C5-T2	C5-T2	C5-T1	C5-T1	C5-T2	C6-T1	C6-T1	C5-T2	C5-T2
*Suprascapularis*	C5-C6	C5-C6	C5-C6	C5-C6	C5-C6	C5-C6	C6-C7	C6	C5-C6	C5-C6
*Subscapularis cranialis*	C5-C6	C5-C6	C6	C6	C5-C6	C5-C6	C6-C7	C6	C5-C6	C5-C6
*Subscapularis medium*	C6	C6	C6-C7	C6-C7	C6-C7	C6-C7	---	---	C6	C6-C7
*Subscapularis caudalis*	C6-C7	C6-C7	C6-C7	C6-C7	C6-C7	C6-C7	C6-C7	C6-C7	C6-C7	C6-C7
*Musculocutaneus*	C6-C7	C6-C7	C6-C7	C6-C7	C6-C7	C6-C7	C6-C7	C6-C7	C6-C7	C6-C7
*Axilaris*	C6-C7	C6-C7	C6-C7	C6-C7	C6-C7	C6-C7	C6-C7	C6-C7	C6-C7	C6-C7
*Radialis*	C6-T1	C6-T1	C7-T1	C7-T1	C7-T1	C7-T1	C6-T1	C6-T1	C6-T1	C6-T1
*Medianus*	C6-T2	C6-T2	C6-T2	C6-T1	C6-T1	C6-T2	C6-T1	C6-T1	C6-T1	C6-T1
*Ulnaris*	C8-T2	C8-T2	C8-T1	C8-T2	C8-T1	C8-T2	C8-T1	C8-T1	C8-T1	C8-T1
*Cutaneus antebrachii caudalis*	C8-T2	C8-T2	T1	C8-T1	T1	T1	T1	T1	T1	T1
*Brachiocephalicus*	C5-C6	C5-C6	C5-C6	C5-C6	C5-C6	C5-C6	C6	C6	C5-C6	C5-C6
*Thoracicus longus*	C7	C7-C8	C7	C7	C7	C7	C7	C7	C7	C7
*Thoracicus lateralis*	C8-T1	C8-T1	C8-T1	C8-T1	C7-T1	C8-T1	C8-T1	C8-T1	C8-T1	C8-T1
*Thoracodorsalis*	C6-T1	C6-C8	C7-C8	C7-C8	C7-C8	C7-C8	C7-C8	C7-C8	C7-C8	C7-C8
*Pectorales craniales*	C6-C7	C6-C7	C7	C7	C7	C7	C7	C7	C6-C7	C6-C7
*Pectorales caudales*	C6-T1	C6-T1	C7-T1	C7-T1	C7-T1	C7-T1	C7-T1	C7-T1	C7-T1	C7-T1

AM: adult male, AF: Adult female, IM: infant male JM: juvenile male, Nn#: number of the specimen, ---: absent.

## References

[1] Nyakatura K., Bininda-Emonds O.R. (2012). Updating the Evolutionary History of Carnivora (Mammalia): A New Species-Level Supertree Complete with Divergence Time Estimates. BMC Biol..

[2] Reid F., Helgen K., González-Maya J.F. Procyon Cancrivorus. https://www.iucnredlist.org/species/41685/45216426.

[3] Emmons L., Helgen K. Nasua nasuai. https://www.iucnredlist.org/species/41684/45216227.

[4] Hassanin A., Veron G., Ropiquet A., Jansen van Vuuren B., Lécu A., Goodman S.M., Haider J., Nguyen T.T. (2021). Evolutionary History of Carnivora (Mammalia, Laurasiatheria) Inferred from Mitochondrial Genomes. PLoS ONE.

[5] Whiteside D.P. (2009). Nutrition and Behavior of Coatis and Raccoons. Vet. Clin. N. Am. Exot. Anim. Pract..

[6] Arispe R., Venegas C., Rumiz D. (2008). Abundancia y Patrones de Actividad Del Mapache (*Procyon cancrivorus*) En Un Bosque Chiquitano de Bolivia. Mastozool. Neotrop..

[7] Hirsch B. (2022). *Nasua nasua* (Ring-Tailed Coati). CABI Compend..

[8] Gatti A., Bianchi R., Rosa C.R.X., Mendes S.L. (2006). Diet of Two Sympatric Carnivores, *Cerdocyon thous* and *Procyon cancrivorus*, in a Restinga Area of Espirito Santo State, Brazil. J. Trop. Ecol..

[9] Quintela F.M., Iob G., Artioli L.G.S. (2014). Diet of Procyon Cancrivorus (Carnivora, Procyonidae) in Restinga and Estuarine Environments of Southern Brazil. Iheringia. Série Zool..

[10] Alves-Costa C.P., Fonseca G., Christófaro C. (2004). Variation in the Diet of the Brown-Nosed Coati (*Nasua nasua*) in Southeastern Brazil. J. Mammal..

[11] Beisiegel B.M. (2001). Notes on the Coati, *Nasua nasua* (Carnivora: Procyonidae) in an Atlantic Forest Area. Braz. J. Biol..

[12] Hirsch B.T. (2009). Seasonal Variation in the Diet of Ring-Tailed Coatis (*Nasua nasua*) in Iguazu, Argentina. J. Mammal..

[13] Campos Z., Mourão G. (2015). Camera Traps Capture Images of Predators of Caiman Crocodilus Yacare Eggs (Reptilia: Crocodylia) in Brazil’s Pantanal Wetlands. J. Nat. Hist..

[14] Desbiez A.L., Borges P.A. (2010). Density, Habitat Selection and Observations of South American Coati Nasua Nasua in the Central Region of the Brazilian Pantanal Wetland. Small Carniv. Conserv..

[15] Olifiers N., de Bianchi R.C., de Mourão G.M., Gompper M.E. (2009). Construction of Arboreal Nests by Brown-Nosed Coatis, *Nasua nasua* (Carnivora: Procyonidae) in the Brazilian Pantanal. Zoologia.

[16] Nowak R.M. (2005). Walker’s Carnivores of the World.

[17] McClearn D. (1992). Locomotion, Posture, and Feeding Behavior of Kinkajous, Coatis, and Raccoons. J. Mammal..

[18] Santos C.M.D., Santos S.M.D., Pizzutto C.S., Custódio A.E.I. (2015). Enriquecimento Ambiental Para Guaxinim, *Procyon cancrivorus* (Cuvier, 1798). Biosci. J..

[19] Demczuk Thomas L., Piccoli R.J., Quintana Bernardi P.E., Sinotti J.F., Andrade Silva V., Fucks de Souza C., Bono Fukushima F. (2021). Braquial Plexus Block and Lumbosacral Epidural in a South American Coati (*Nasua nasua*). Acta Sci. Vet..

[20] Pinheiro L.L., Branco É., Souza D.C., Pereira L.H.C., Lima A.R. (2014). Descrição Do Plexo Braquial Do Cachorro-Do-Mato (*Cerdocyon thous* Linnaeus, 1766). Ciência Anim. Bras..

[21] Chagas K.L.S., Moura Fé L.C., Pereira L.C., Lima A.R., Branco É. (2014). Descrição Morfológica Do Plexo Braquial de Jaguatirica (*Leopardus pardalis*). Biotemas.

[22] Haligur A., Ozkadif S. (2021). Macroanatomical Investigation of the Plexus Brachialis in the Red Fox (*Vulpes vulpes*). Pak. J. Zool..

[23] Souza Junior P., da Carvalho N.C., Medeiros-do-Nascimento R., de Dantas P.O., Bernardes F.C.S., Abidu-Figueiredo M. (2022). Brachial Plexus Formation in Jaguarundi (*Puma yagouaroundi*). Anat. Histol. Embryol..

[24] Sánchez H.L., Silva L.B., Rafasquino M.E., Mateo A.G., Zuccolilli G.O., Portiansky E.L., Alonso C.R. (2013). Anatomical Study of the Forearm and Hand Nerves of the Domestic Cat (*Felis catus*), Puma (*Puma concolor*) and Jaguar (*Panthera onca*). Anat. Histol. Embryol..

[25] Pinheiro L.L., Branco É.R., De Souza D.C., De Souza A.C.B., Pereira L.C., Lima A.R. (2013). De Descrição Do Plexo Braquial Do Cachorro-Do-Mato-de-Orelhas-Curtas (*Atelocynus microtis*—Sclater, 1882): Relato de Caso. Biotemas.

[26] De Souza D.A.S., de Castro T.F., Franceschi R.D.C., Silva Filho R.P., Pereira M.A.M. (2010). Formação Do Plexo Braquial e Sistematização Dos Territórios Nervosos Em Membros Torácicos de Lobos-Marinhos *Arctocephalus australis*. Braz. J. Vet. Res. Anim. Sci..

[27] Barreto-Mejía R., Ceballos C.P., Tamayo-Arango L.J. (2022). Anatomical Description of the Origin and Distribution of the Brachial Plexus to the Antebrachium in One Puma (*Puma concolor*) (Linnaeus, 1771). Anat. Histol. Embryol..

[28] Hakkı Nur İ., Keleş H., Pérez W. (2020). Origin and Distribution of the Brachial Plexus of the Van Cats. Anat. Histol. Embryol..

[29] Roos H., Vollmerhaus B. (2005). Konstruktionsprinzipien an Der Vorder- Und Hinterpfote Der Hauskatze (*Felis catus*). 4. Mitteilung: Muskelinnervation Und Bewegungsanalyse+. Anat. Histol. Embryol..

[30] Enciso-García L.M., Vélez-García J.F. (2022). Origin and Distribution of the Brachial Plexus in Kinkajou (*Potos flavus*—Schreber, 1774). Anat. Histol. Embryol..

[31] Grzeczka A., Zdun M. (2022). The Structure of the Brachial Plexus in Selected Representatives of the Caniformia Suborder. Animals.

[32] Souza-Junior P., Wronski J.G., Carvalho N.C., Abidu-Figueiredo M. (2018). Brachial Plexus in the *Leopardus geoffroyi*. Ciência Anim. Bras..

[33] International Committee on Veterinary Gross Anatomical Nomenclature, World Association of Veterinary Anatomists (2017). Nomina Anatómica Veterinaria.

[34] Hermanson J., Evans H., De Lahunta A., Hermanson J.W., Evans H., De Lahunta A. (2020). Miller and Evan’s Anatomy of the Dog.

[35] Davis D.D. (1964). The Giant Panda: A Morphological Study of Evolutionary Mechanisms. Fieldiana.

[36] Langworthy O.R. (1924). The Panniculus Carnosus in Cat and Dog and Its Genetical Relation to the Pectoral Musculature. J. Mammal..

[37] Vélez García J.F., Miglino M.A. (2022). Evolutionary Comparative Analysis of the Extrinsic Thoracic Limb Muscles in Three Procyonids (*Procyon cancrivorus* Cuvier, 1798, *Nasua nasua* Linnaeus, 1766, and *Potos flavus* Schreber, 1774) Based on Their Attachments and Innervation. Anat. Sci. Int..

[38] Barone R. (2020). Anatomie Comparée Des Mammifères Domestiques. Tome 2: Arthrologie et Myoulogie.

[39] Perdomo-Cárdenas V., Patiño-Holguín C., Vélez-García J.F. (2021). Evolutionary and Terminological Analysis of the Flexor Digitorum Superficialis, Interflexorii and Palmaris Longus Muscles in Kinkajou (*Potos flavus*) and Crab-Eating Racoon (Procyon Cancrivorus). Anat. Histol. Embryol..

[40] Allam M.W., Lee D.G., Nulsen F.E., Fortune E.A. (1952). The Anatomy of the Brachial Plexus of the Dog. Anat. Rec..

[41] Aubert L., Carozzo C., Devillaire A.-C., Crevier-Denoix N., Moissonnier P. (2004). Macro- and Microanatomical Characterization of the Cat Brachial Plexus. Cells Tissues Organs.

[42] Singh B. (2018). Dyce, Sack and Wensing’ Textbook of Veterinary Anatomy.

[43] Demiraslan Y., Aykut M., Özgel Ö. (2015). Macroanatomical Characteristics of Plexus Brachialis and Its Branches in Martens (*Martes foina*). Turk. J. Vet. Anim. Sci..

[44] Backus T.C., Solounias N., Mihlbachler M.C. (2016). The Brachial Plexus of the Sumatran Rhino (Dicerorhinus Sumatrensis) and Application of Brachial Plexus Anatomy Toward Mammal Phylogeny. J. Mamm. Evol..

[45] Souza-Junior P., Carvalho N.C., Mattos K., Santos A.L.Q. (2014). Origens e Ramificações Do Plexo Braquial No Cachorro-Do-Mato *Cerdocyon thous* (Linnaeus, 1766). Pesqui. Vet. Bras..

[46] Souza-Junior P., da Cruz de Carvalho N., de Mattos K., Abidu Figueiredo M., Luiz Quagliatto Santos A. (2017). Brachial Plexus in the Pampas Fox (*Lycalopex gymnocercus*): A Descriptive and Comparative Analysis. Anat. Rec..

[47] Silva L.B., Sánchez H.L. (2013). La Inervación Del Miembro Torácico En Felinos. AnAlectA Vet..

[48] Felipe R., Silva F., França G.L., Silva E.M., Leonel L.C., Carvalho-Barros R.A., Silva D.C., Silva Z. (2014). Anatomia Descritiva Do Nervo Musculocutâneo Em Quatis (*Nasua nasua*, Linnaeus, 1766). Enciclopédia Biosf..

[49] Vélez-García J.F., Patiño-Holguín C., Duque-Parra J.E. (2018). Anatomical Variations of the Caudomedial Antebrachial Muscles in the Crab-Eating Fox (*Cerdocyon thous*). Int. J. Morphol..

[50] Reighard J., Jennings H.S. (1901). Anatomy of the Cat.

[51] König H.E. (1992). Anatomie Der Katze: Mit Hinweisen Für Die Tierärztliche Praxis.

[52] Hudson L.C., Hamilton W.C. (2010). Atlas of Feline Anatomy for Veterinarians.

[53] König H., Mülling C., Seeger J., Liebich H., König H.E., Liebich H.G. (2020). Nervous System (Systema Nervosum). Veterinary Anatomy of Domestic Animals: Textbook and Colour Atlas.

[54] Arłamowska-Palider A. (1970). Morphological Studies on the Main Branches of the Radial Nerve in Mammals. Acta Theriol..

[55] Hermanson J., Hermanson J.W., Evans H., De Lahunta A. (2020). The Muscular System. Miller’s Anatomy of the Dog.

[56] Kamali Y. (2022). Aberrant Arrangement of the Musculocutaneous and Median Nerves in the Thoracic Limbs of a Mixed-breed Dog Cadaver. Anat. Histol. Embryol..

[57] Vélez García J.F., Ospina Orozco A., Duque Parra J.E. (2018). Origen de Los Nervios Del Plexo Braquial Del Venado Coliblanco (*Odocoileus virginianus*) En Comparación Con Otros Rumiantes. Rev. Investig. Vet. Del Perú.

[58] Arłamowska-Palider A. (1970). Comparative Anatomical Studies of Nervus Musculocutaneus in Mammals. Acta Theriol..

[59] Kawashima T., Thorington R.W., Bohaska P.W., Chen Y.-J., Sato F. (2015). Anatomy of Shoulder Girdle Muscle Modifications and Walking Adaptation in the Scaly Chinese Pangolin (*Manis pentadactylapPentadactyla*: Pholidota) Compared with the Partially Osteoderm-Clad Armadillos (Dasypodidae). Anat. Rec..

[60] Upham N.S., Esselstyn J.A., Jetz W. (2019). Inferring the Mammal Tree: Species-Level Sets of Phylogenies for Questions in Ecology, Evolution, and Conservation. PLoS Biol..

[61] Liebich H.G., Maierl J., König H.E., König H.E., Liebich H.G. (2020). Forelimbs or Thoracic Limbs (Membra Thoracica). Veterinary Anatomy of Domestic Animals: Textbook and Colour Atlas.

[62] Windle B., Parsons F. (1897). Myology of the Terrestrial Carnivora. Part I. Muscles of the Head, Neck, and Fore-Limb. Proc. Zool. Soc. Lond..

[63] Abdala V., Diogo R. (2010). Comparative Anatomy, Homologies and Evolution of the Pectoral and Forelimb Musculature of Tetrapods with Special Attention to Extant Limbed Amphibians and Reptiles. J. Anat..

[64] Vélez-García J.F., Arbeláez-Quiñones A.C., Montealegre-Hurtado K.D. (2021). Evolutionary Adaptations in the Flexor Digitorum Profundus Muscle in *Tamandua mexicana* (Xenarthra, Myrmecophagidae). Anat. Rec..

[65] Niederschuh S.J., van Beesel J., Schmidt M. (2022). The Role of Sensory Feedback from Carpal Sinus Hairs in Locomotor Kinematics of Rats (*Rattus norvegicus*, Rodentia) during Walking on Narrow Substrates. Zoology.

[66] Fundin B.T., Arvidsson J., Rice F.L. (1995). Innervation of Nonmystacial Vibrissae in the Adult Rat. J. Comp. Neurol..

[67] Bailey C.S., Kitchell R.L., Johnson R.D. (1982). Spinal Nerve Root Origins of the Cutaneous Nerves Arising from the Canine Brachial Plexus. Am. J. Vet. Res..

[68] Numata N., Kida M.Y., Kudoh H. (1996). Ramification Patterns of the Nerves Innervating the Forearm Extensors in Mammals and Reptiles. Okajimas Folia Anat. Jpn..

[69] Koizumi M. (2022). Comparative Anatomy of the Subscapularis, Teres Major and Latissimus Dorsi Muscles from Salamanders to Mammals with Special Reference to Their Innervations from the Brachial Plexus. Anat. Sci. Int..

[70] Vélez-García J.F., Chunganá-Caicedo D., Saavedra-Montealegre S. (2022). Gross Anatomy of the Craniolateral Antebrachial Muscles in Kinkajou (*Potos flavus*, Carnivora): Intra- and Interspecific Variants within the Family Procyonidae. Anat. Histol. Embryol..

[71] Vélez-García J.F., Marín-González L., Monroy-Cendales M.J., Miglino M.A. (2022). Craniolateral Forearm Muscles of the Crab-Eating Raccoon (*Procyon cancrivorus*) and a Comparative Review with Other Carnivorans. Iheringia. Série Zool..

[72] Echeverry J., Vélez J., Sánchez C. (2015). Descripción Anatómica de Los Músculos Cráneo-Laterales Superficiales Del Antebrazo Del Zorro Perruno (*Cerdocyon thous*). Rev. Colomb. Cienc. Anim..

[73] Diogo R., Abdala V. (2010). Muscles of Vertebrates: Comparative Anatomy, Evolution, Homologies and Development.

[74] Koizumi M., Sakai T. (1997). On the Morphology of the Brachial Plexus of the Platypus (*Ornithorhynchus anatinus*) and the Echidna (*Tachyglossus aculeatus*). J. Anat..

[75] Turnbull B.G., Rasmusson D.D. (1986). Sensory Innervation of the Raccoon Forepaw: 1. Receptor Types in Glabrous and Hairy Skin and Deep Tissue. Somatosens. Res..

[76] Munger B.L., Pubols L.M. (1972). The Sensorineural Organization of the Digital Skin of the Raccoon (Part 1 of 2). Brain. Behav. Evol..

